# Biomimetic Fibrinogen Nanofiber Scaffolds for Vascular Hematopoietic Stem Cell Niche Engineering

**DOI:** 10.1002/adhm.202503449

**Published:** 2025-10-28

**Authors:** Sophia Lena Meermeyer, Arundhati Joshi, Constantin von Kaisenberg, Dorothea Brüggemann, Cornelia Lee‐Thedieck

**Affiliations:** ^1^ Institute of Cell Biology and Biophysics Leibniz University Hannover Herrenhäuser Str. 2 30149 Hannover Germany; ^2^ Institute for Biophysics University of Bremen Otto‐Hahn‐Allee NW1 28359 Bremen Germany; ^3^ Hannover Medical School Department for Obstetrics Gynecology and Reproductive Medicine Carl‐Neuberg‐Str. 1 30625 Hannover Germany; ^4^ Biophysics and Applied Biomaterials City University of Applied Sciences Bremen Neustadtswall 30 28199 Bremen Germany

**Keywords:** basement membrane mimetic, endothelial cells, hematopoietic stem and progenitor cells, in vitro model, mesenchymal stem and stroma cells, nanofibrous fibrinogen scaffolds, vascular stem cell niche

## Abstract

Hematopoietic stem cells (HSCs) can reconstitute the human blood system. In vivo, HSCs are localized in and regulated by distinct bone marrow (BM) microenvironments, or niches, like the vascular HSC niches near fenestrated sinusoidal blood vessels. These delicate structures, comprising a single‐layered endothelium and a discontinuous basement membrane, pose challenges in soft tissue engineering. In this study, the basement membrane in vascular niches is mimicked using nanofibrous fibrinogen scaffolds. A novel clamping system enables handling the scaffold as a membrane and seeding both sides−one with microvascular endothelial cells (HMEC‐1) and the other with mesenchymal stem and stroma cells (iMSC#3). Subsequently, HSCs and their progenitors (HSPCs) are introduced from both sides to emulate their niche dynamics (residency, exit, and homing). The study reveals that the fibrinogen scaffolds are highly cytocompatible and show good cell‐adhesive properties. In addition, HSPCs are able to migrate through the scaffolds, validating them as fenestrated basement membrane mimetics. This in vitro model offers insights into HSPC behavior in the vascular niche and can serve as a drug testing platform in future studies. Moreover, beyond HSCs, the presented scaffold‐based mimetic of the basement membrane offers new opportunities for mimicking and studying vasculature in tissue engineering approaches.

## Graphical abstract

This study presents an advanced in vitro model of the vascular hematopoietic stem cell niche using self‐assembled fibrinogen nanofibers, mimicking the basement membrane in bone marrow (BM) sinusoids. The model supports the coculture of microvascular endothelial cells, stromal cells, and hematopoietic stem and progenitor cells, providing insights into niche dynamics and offering potential for drug testing and tissue engineering applications.

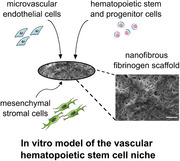

## 1 Introduction

Hematopoietic stem cells (HSCs) have the ability to self‐renew and give rise to all types of blood cells. Due to these properties, HSCs maintain the hematopoietic system and continuously replenish the human body.^[^ ^1^, ^2^ ^]^ During hematopoiesis, the process of generating mature blood cells, HSCs differentiate into multipotent progenitors, which only have limited self‐renewal potential and can give rise to progenitors of the erythroid, myeloid and lymphoid lineage.^[^ ^1^, ^3^, ^4^ ^]^ Collectively, these cells can be referred to as hematopoietic stem and progenitor cells (HSPCs).

The major site of adult hematopoiesis is the bone marrow (BM),^[^ ^4^, ^5^, ^6^ ^]^ where HSCs reside in specific niches, a concept first proposed in 1978.^[^ ^7^ ^]^ These specialized microenvironments are crucial for HSC maintenance and targeted differentiation.^[^ ^5^, ^8^, ^9^ ^]^ Although the exact localization of HSCs in the BM and the definition of HSC niches remain a matter of ongoing research,^[^ ^10^, ^11^ ^]^ two HSC niches are commonly described: the endosteal niche at the inner surface (endosteum) of trabecular bones and the vascular niche in close proximity to blood vessels. Recent studies revealed that the majority of the HSCs can be found in the vascular niche,^[^ ^5^, ^12^, ^13^ ^]^ which can be further subdivided into arteriolar and sinusoidal niches^[^ ^13^, ^14^ ^]^ named after the type of blood vessel they are close to. Within the different niches, supportive cells, soluble factors, oxygen tension, and composition as well as stiffness of the extracellular matrix (ECM) contribute to the niche environment and thereby regulate HSC fate.^[^ ^9^, ^15^ ^]^ Whereas the endosteal and arteriolar niches are classically assumed to promote quiescence and maintenance, the sinusoidal niche environment is mainly considered to promote HSC activation, proliferation, migration, and differentiation.^[^ ^13^, ^16^, ^17^, ^18^ ^]^ However, there is increasing evidence that a strict separation of the niches and their assigned function is not reasonable and that the niches can promote both, HSC quiescence and activation depending on the physiological state.^[^ ^19^, ^20^, ^21^ ^]^

Given the spatial proximity of endosteal and arteriolar niches and their similar influence on HSPCs, many studies only differentiate between endosteal and vascular niches, with the latter being referred to as the sinusoidal vascular niche. Accordingly, the term vascular niche is used in the current study to describe the microenvironment at sinusoidal vessels, which consist of a single‐layered, fenestrated endothelium enclosed by an equally fenestrated basement membrane.^[^ ^22^ ^]^ These fenestrations result in a higher leakiness, which facilitates migration and egress of cells from the BM into the blood stream and vice versa.

Since the BM niches are highly complex microenvironments, HSCs rapidly differentiate and lose their stem cell properties when cultured on standard tissue culture polystyrene (TCPS).^[^ ^23^, ^24^ ^]^ Thus, in vitro models that closely resemble in vivo niches are needed for fundamental research as well as for various applications such as tissue engineering or drug development. Over the last decades, the number of studies that developed models to mimic the BM microenvironment has greatly increased.^[^ ^15^, ^25^ ^]^ These models have evolved from two‐dimensional (2D) coculture studies that investigate mutual interactions of different niche cell types and HSPCs in a simplified system,^[^ ^26^, ^27^, ^28^ ^]^ toward three‐dimensional (3D) culture using biomaterials such as hydrogels or scaffolds,^[^ ^29^, ^30^, ^31^ ^]^ up to complex experimental setups in microfluidic chips (BM‐on‐a‐chip models)^[^ ^32^, ^33^, ^34^ ^]^ or BM organoids.^[^ ^35^, ^36^ ^]^ Since the majority of HSPCs was found to be located in the vascular niche,^[^ ^5^, ^12^, ^13^ ^]^ accurately mimicking this particular microenvironment in vitro is highly important.

A suitable in vitro model should balance complexity and simplicity to properly resemble the in vivo situation. To mimic the vascular niche, cocultures with mesenchymal stem and stromal cells (MSCs) and endothelial cells (ECs) were often used, as these are the major supporter cell types of HSPCs in this niche in vivo.^[^ ^33^, ^34^, ^37^ ^]^ In addition, the culture medium is often supplemented with cytokines to ensure cell survival and preserve the HSPCs from exhaustion. Most studies use a mixture of stem cell factor (SCF), FMS‐like tyrosine kinase 3 ligand (Flt‐3 ligand), thrombopoietin (TPO), interleukin‐3 (IL‐3), and interleukin‐6 (IL‐6) as these cytokines were shown to have positive effects on the expansion of HSPCs.^[^ ^38^, ^39^ ^]^ Besides, several in vitro models contain ECM‐like structures as the availability of cellular adhesion cues was shown to influence HSC fate.^[^ ^40^, ^41^ ^]^

One way of incorporating ECM‐like structures into in vitro models is the use of nanofibers, which possess advantageous properties such as high porosity, variable pore‐size distribution, and high surface‐area‐to‐volume ratios.^[^ ^42^, ^43^ ^]^ As nanofibers also provide structural and spatial cues, they can be termed as a stepping‐stone from 2D to 3D culture.^[^ ^31^ ^]^ Nanofibers can be fabricated from various materials. Synthetic materials such as polycaprolactone (PCL) or polylactic acid (PLA) offer good mechanical qualities and high biostability, but often require cell adhesive coatings and cannot be remodeled by cells.^[^ ^44^ ^]^ Thus, natural polymers are more advantageous for the use in in vitro models. Techniques for fabrication of nanofibers include electrospinning, self‐assembly, and phase separation.^[^ ^44^, ^45^ ^]^

One advantage of self‐assembly is that the fiber fabrication can be performed in physiological media, whereas both other techniques involve organic solvents. Residues of these solvents that remain in the fibers can impair cell survival.^[^ ^46^, ^47^ ^]^ In addition, the high concentrations^[^ ^48^, ^49^ ^]^ and electric fields^[^ ^50^ ^]^ required for electrospinning can affect the biological function of the protein.^[^ ^46^ ^]^ The self‐assembly method uses the ability of molecules to form organized patterns or structures through noncovalent forces, yielding very small nanofibers that closely mimic ECM‐like structures.^[^ ^46^, ^51^ ^]^ Self‐assembly usually occurs spontaneously, but it can also be induced by certain stimuli such as light,^[^ ^52^ ^]^ enzymatic reactions,^[^ ^53^ ^]^ pH change,^[^ ^54^ ^]^ or ionic strength.^[^ ^55^ ^]^ Although many proteins can self‐assemble, predicting and regulating the process in order to obtain thin yet stable scaffolds is challenging.^[^ ^56^ ^]^ Since fibrinogen exhibits cell adhesion motifs, is highly biocompatible as well as biodegradable,^[^ ^57^ ^]^ and self‐assembled fibrinogen nanofibers with the required properties can be reproducibly fabricated,^[^ ^54^, ^55^, ^58^, ^59^ ^]^ these scaffolds were used in the current study to mimic the basement membrane.

Developing an in vitro model that fully replicates the complexity of the vascular niche remains challenging. Although a number of studies already successfully replicated relevant aspects of the vascular niche,^[^ ^30^, ^33^, ^34^, ^36^, ^37^, ^60^, ^61^, ^62^, ^63^, ^64^ ^]^ most of the in vitro models so far did not consider to resemble the basement membrane and used human umbilical vein endothelial cells (HUVECs) instead of microvascular or sinusoidal ECs. In this way, tight vessel structures were created, which differ from the thin‐walled, fenestrated vessels characteristic for BM sinusoids. The aim of this study was therefore to develop a vascular niche model, which reflects the delicate basement membrane structure and the microvascular nature of ECs in BM sinusoids. For this purpose, nanofibrous fibrinogen scaffolds were applied as a platform to coculture HSPCs, microvascular ECs, and MSCs. HSPCs were isolated from human umbilical cord blood (UCB) and the cell lines HMEC‐1 and iMSC#3 were used as microvascular EC and MSC models, respectively. One side of the biomaterial was seeded with HMEC‐1 cells (EC side) and the other side with iMSC#3 cells (MSC side) to resemble the inner and outer layers of the sinusoidal vessel. Subsequently, HSPCs were introduced from either the MSC‐ or the EC‐seeded side to mimic the release into the blood stream or the homing to the niche.

The developed model was used to investigate the influence of microvascular ECs, MSCs, and the scaffold material on HSPC proliferation and differentiation. In addition, transmigration behavior of HSPCs was analyzed to assess the suitability of the scaffold as fenestrated basement membrane mimetic.

## 2 Results

### 2.1 Nanofibrous Fibrinogen Scaffolds

**Figure 1:**
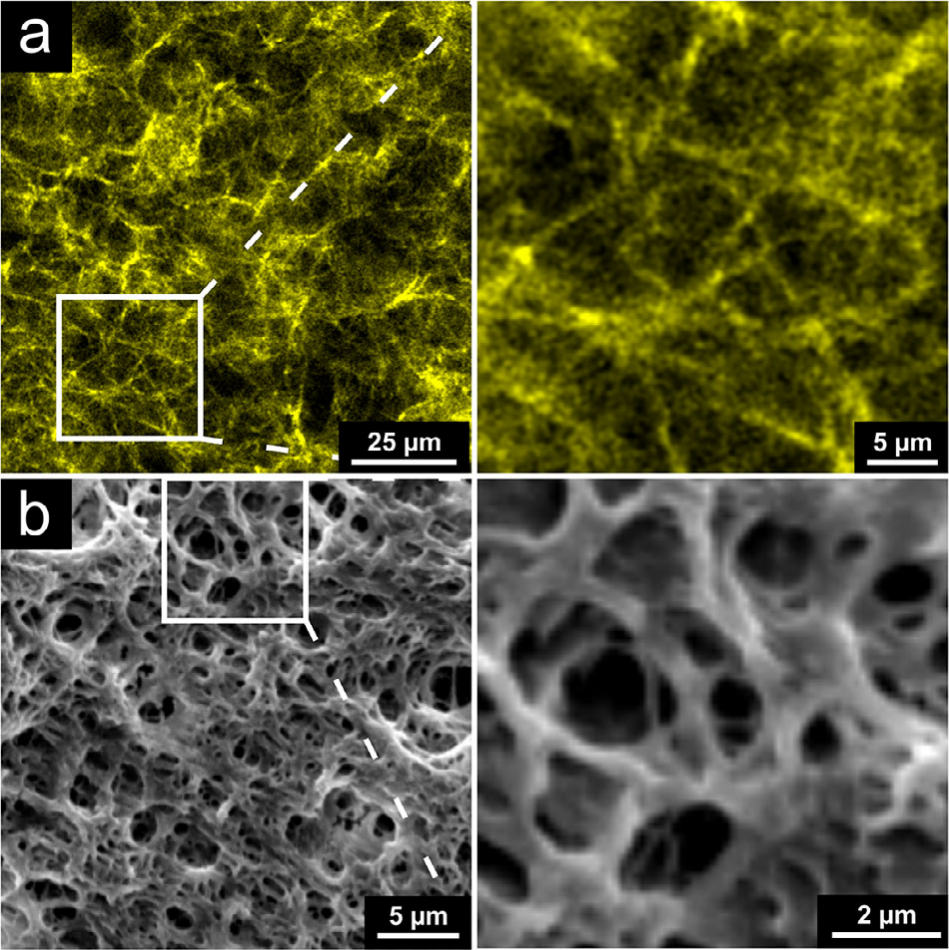
Topography and porous architecture of nanofibrous fibrinogen scaffolds. a) The fibrous structure of the scaffolds was visualized by the strong autofluorescence after excitation with a 488 nm laser, which was detected using a confocal laser‐scanning microscope (cLSM) (10× objective, Airyscan detector, multiplex mode (SR 4Y)). b) The scanning electron microscopy (SEM) image of an immobilized, dried fibrinogen scaffold confirmed the porous and nanofibrous network architecture. Imaging was performed with a Quanta 200 SEM from FEI (SE detector, working distance: 9‐10 mm, operation voltage: 8 kV).

The fibrinogen scaffolds were fabricated using salt‐induced self‐assembly in the presence of 2.5× phosphate‐buffered saline.^[^ ^55^, ^65^ ^]^ The fabrication process was first described by Stapelfeldt et al.^[^ ^55^ ^]^ in 2019 and the scaffolds were characterized extensively in several studies.^[^ ^55^, ^58^, ^59^, ^65^ ^]^ When dry, fibrinogen scaffolds had a thickness of 2.7 ± 0.4 µm with nanofiber diameters around 230 nm that swelled to approximately 340 nm upon rehydration in DMEM cell culture medium.^[^ ^59^ ^]^ Cross‐sectional analysis showed average pore sizes of 20 ± 9 µm.^[^ ^65^ ^]^ Based on these features, self‐assembled fibrinogen scaffolds were chosen to mimic the fenestrated and fibrous network architecture of the basement membrane. In the current study, fibrinogen scaffolds that were immobilized on thin glass coverslips were used for initial cell adhesion studies and for scanning electron microscopy (SEM) analyses. For the in vitro model, free‐standing fibrinogen scaffolds were clamped, which enabled the double‐sided seeding with different cell types of the vascular hematopoietic stem cell niche. The scaffolds possessed a strong autofluorescence after excitation with a 488 nm laser, which enabled the visualization of the fibrous architecture via microscopy (**Figure** **1**a). Furthermore, the nanofibers and pores were visualized by SEM analysis (Figure 1b).

### 2.2 Cell Adhesion to Nanofibrous Fibrinogen Scaffolds

**Figure 2:**
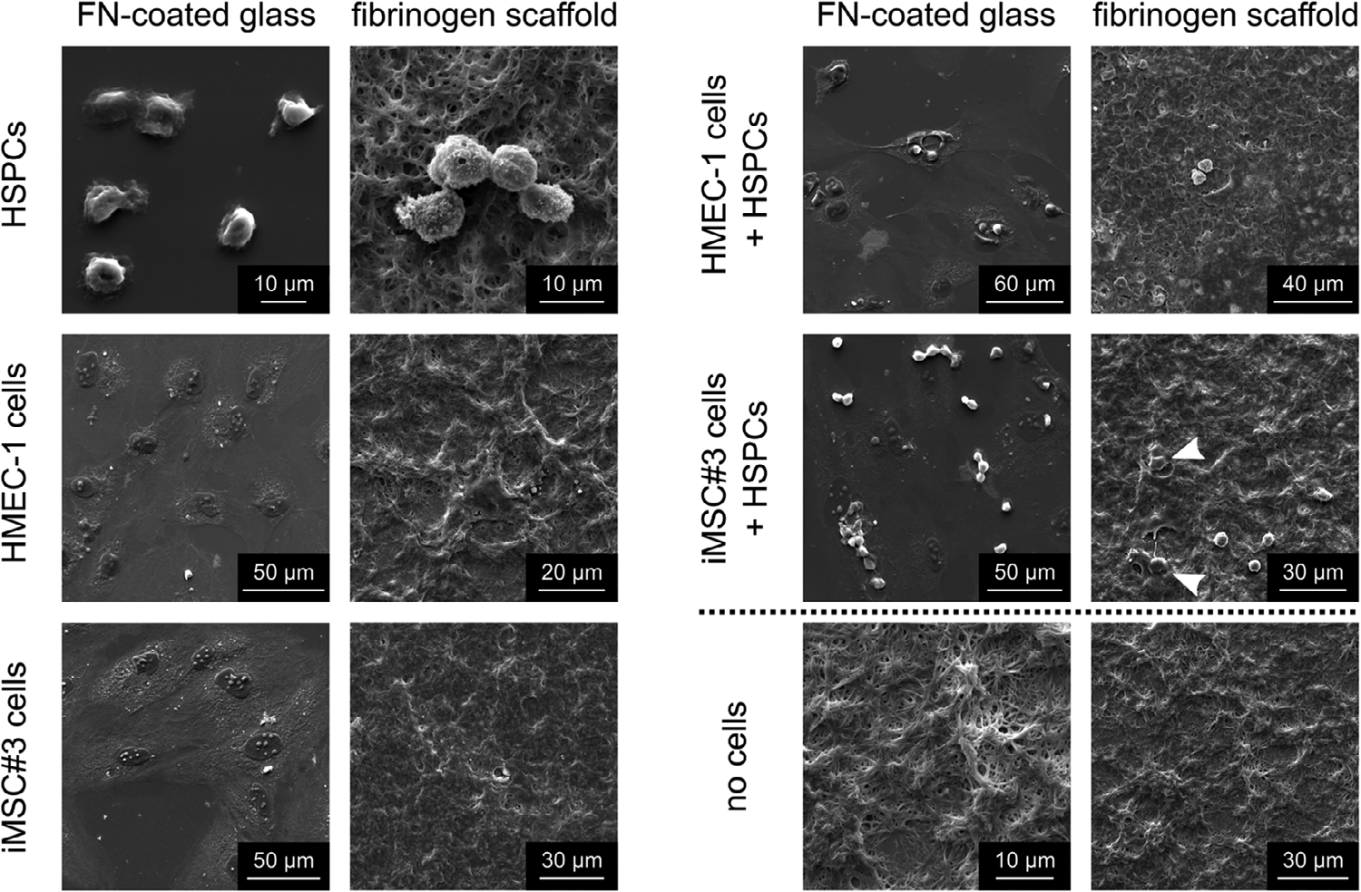
Adhesion of HSPCs, HMEC‐1, and iMSC#3 cells to immobilized fibrinogen scaffolds in comparison to adhesion to fibronectin (FN) coated glass coverslips visualized by representative scanning electron microscopy (SEM) images. The culture conditions are described on the left and the surface material is described on top of the images. White arrowheads mark HSPCs that are located underneath the iMSC#3 cells. Imaging was performed on a Quanta 200 SEM from FEI (SE detector, working distance: 9–10 mm, operation voltage: 15 kV (FN‐coated glass), and 8 kV (fibrinogen scaffolds), *n* ≥ 6 per condition).

To initially assess the suitability of the nanofibrous fibrinogen scaffolds for the establishment of vascular niche models, their ability to interact with the different cell types was investigated. For this purpose, morphology, adhesion and interactions of HSPCs, HMEC‐1 (ECs) and iMSC#3 cells on fibrinogen scaffolds were analyzed via SEM as well as immunofluorescence (IF) staining. For these experiments, scaffolds immobilized on glass coverslips were used, with fibronectin (FN) coated coverslips serving as a cell‐adhesive positive control for comparison. SEM images (**Figure** **2**) show that all cells adhered well to both surfaces, exhibited normal cell morphologies, and closely interacted with the scaffolds. HMEC‐1 and iMSC#3 cells spread thin and flat on FN‐coated glass with slightly elevated nuclei. On the scaffolds, individual cells were harder to distinguish due to the undulated fiber surface and dense growth. In addition, HMEC‐1 and iMSC#3 cells protruded into the scaffolds in such a way that the cell edges were barely visible. However, the cell‐grown scaffolds clearly differed from the bare scaffold surface where the nanofibers were clearly visible. HSPCs displayed round or slightly polarized (round with small protrusions) morphologies on both materials. On FN‐coated glass, pronounced HSPC adhesion structures such as lamellipodia, small filopodia, or uropods were observed, whereas these were barely visible on the scaffolds due to the fibrous surface structure. In the cocultures, HSPCs were often localized on top of HMEC‐1 and iMSC#3 cells. In the image of the iMSC#3 coculture on the scaffold, it can be seen that some HSPCs were located beneath iMSC#3 cells (Figure 2, white arrowheads).

**Figure 3:**
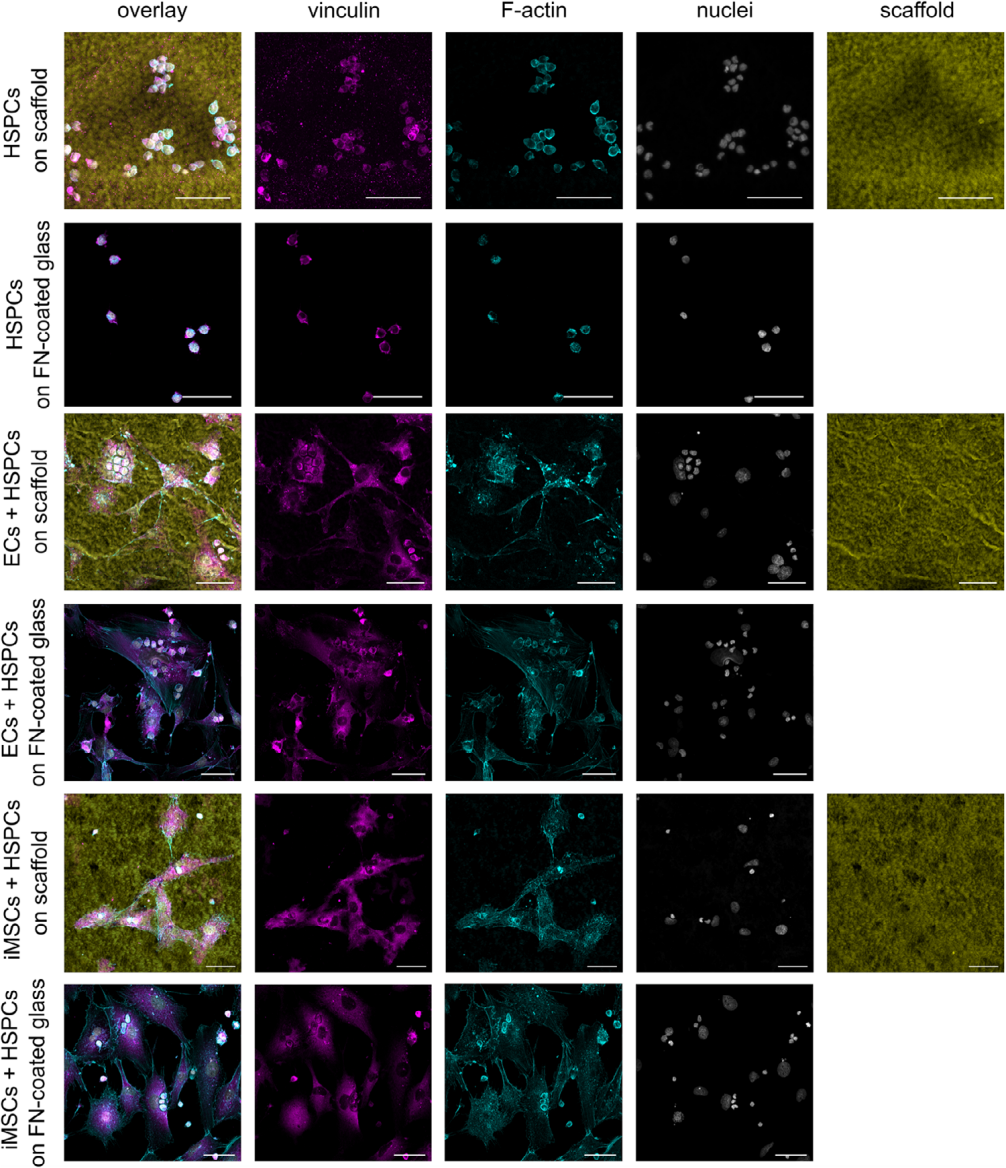
Adhesion of HSPCs, HMEC‐1 (ECs), and iMSC#3 cells (iMSCs) to immobilized fibrinogen scaffolds in comparison to adhesion to FN‐coated glass coverslips visualized by vinculin staining. The cells were cultured for a period of 4 days, subsequently IF stained and analyzed via microscopy. *Z*‐stacks of the culture conditions (described on the left) were captured (4 per condition) and maximum projection images were created. Images of the different channels are depicted in columns. In the first column, overlay images of all channels are given for every culture condition, followed by single channel images of the vinculin staining (pink), F‐actin staining (turquoise), nucleus staining (white), and scaffold autofluorescence (yellow). The images were taken with a confocal laser‐scanning microscope (cLSM) in the 20× objective using the Airyscan detector in the multiplex mode (CO‐8Y for cultures on scaffolds, SR‐4Y for cultures on FN‐coated glass). The scale bars represent 50 µm.

The IF staining confirmed the observations from SEM analyses and provided additional insights into cell morphology and adhesion. Furthermore, the fluorescent staining made it possible to visualize the protrusion of the cells into the scaffolds. HSPCs exhibited a round or slightly polarized morphology (round with small protrusions) on both surfaces and vinculin was evenly distributed at the HSPC peripheries (**Figure** **3**). In addition, HSPCs were mainly found in cell clusters in the monocultures. For HMEC‐1 (indicated as ECs in Figure 3) and iMSC#3 cells (indicated as iMSCs in Figure 3), differences between the adhesion to the scaffold and the FN‐coated glass coverslips were visible. Both cell types displayed less defined F‐actin stress fibers, smaller cell spreading areas, and more finger‐like protrusions on fibrinogen scaffolds compared to FN‐coated glass (Figure 3). Using these protrusions, HMEC‐1 and iMSC#3 cells grew into the scaffolds. These observations are supported by the analysis of the cell spreading area and the solidity of the cells on both materials, which were performed on IF images of single cultures (HSPCs: Figure 3, HMEC‐1 + iMSC#3 cells: Figure S1, Supporting Information). The quantification analysis revealed a significant larger cell spreading area of all three cell types on the flat compared to the nanofibrous surface (Figure S2a–c, Supporting Information). The observed difference was most pronounced for the iMSC#3 cells, followed by HMEC‐1 cells. For the HSPCs, the least difference between the cell spreading area on both materials was found (Figure S2a–c, Supporting Information). In addition, a slightly lower cell solidity was observed for all three cell types on the immobilized fibrinogen scaffolds (Figure S2d–f, Supporting Information) indicating fewer or smaller protrusions compared to the FN‐coated glass. The lower cell solidity was significant for the HMEC‐1 cells and only slightly apparent for iMSC#3 cells and HSPCs (Figure S2d–f, Supporting Information). Moreover, Vinculin, a key mediator of cellular adhesion,^[^ ^66^, ^67^ ^]^ was diffusely located throughout the cells and distinct vinculin dots were observable close to the nucleus as well as at the tips of F‐actin bundles (see Figure 3). These vinculin structures were more pronounced in the cells on FN‐coated glass compared to cells on scaffolds due to the strong nano‐microtopography of the scaffolds. Images of negative IF staining controls omitting the primary antibody (AB) can be found in Figure S3 (Supporting Information). In summary, the fibrinogen scaffolds proved to be adhesive for all tested cell types with cells tightly interacting with the nanofibrous structure. Thus, the scaffolds appeared to be suitable for further use as basement membrane mimetic.

### 2.3 Establishment of Fibrinogen Scaffolds as Vascular Niche Model

**Figure 4:**
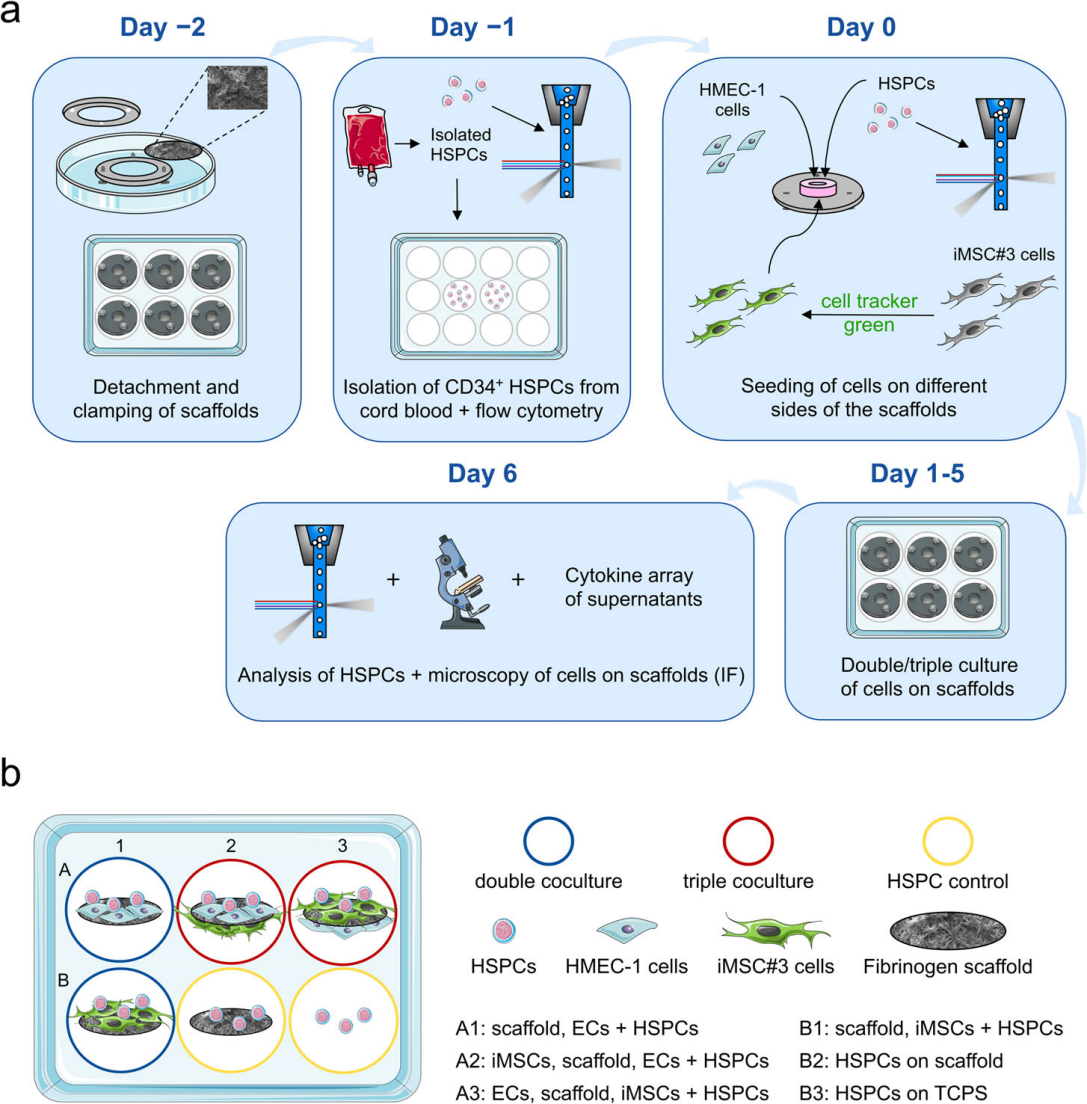
Workflow of experiments and experimental conditions. a) All experiments were performed according to the same workflow. The first step was the detachment and clamping of the free‐standing fibrinogen scaffolds. Next, CD34^+^ HSPCs were isolated from cord blood and tested for CD34 purity (flow cytometry). The next day, HSPCs, HMEC‐1, and iMSC#3 cells were seeded on different sides of the scaffold and cultured in different experimental conditions. iMSC#3 cells were stained with a cell tracker before seeding to identify them in cocultures. After a culture period of 5 days, nonadherent HSPCs were rinsed from the scaffolds and analyzed. In addition, the cell‐seeded scaffolds and the medium supernatants were also analyzed. b) In every experiment, six experimental conditions were compared: two double cocultures (blue: A1, B1), two triple cocultures (red: A2, A3), and two HSPC monoculture controls (yellow: B2, B3). The detailed composition of the conditions is described in the legend and given from bottom to top, with commas between the different layers and a plus indicating cells seeded together onto the same side of the scaffold.

After proving the cell‐adhesive nature of surface‐bound fibrinogen scaffolds, free‐standing scaffolds were tested toward their suitability as basement membrane mimetic. For this purpose, a technique based on clamping the scaffolds between two rings was developed, which enabled the cell seeding from either side (**Figure** **4**). In this way, individual scaffolds served as a nanofibrous membrane between the different cell layers. In every experiment, six experimental conditions were compared: two double cocultures, two triple co cultures and two HSPC monoculture controls (Figure 4b). The composition of the respective conditions is always given from bottom to top, with commas between the different the different layers and a plus indicating cells seeded together onto the same side of the scaffold (e.g., iMSCs, scaffold, ECs + HSPCs describes a scaffold seeded with iMSCs from one side and with ECs and HSPCs from the other side).

After 5 days of culture, the cell‐grown scaffolds were IF stained. CD45 served as a marker for HSPCs, iMSC#3 cells were detectable due to a staining with cell tracker green (CTG) and HMEC‐1 cells could be identified as these were the only cells stained with phalloidin alone. In this way, microscopy enabled to study the localization of the different cell types, cell–cell as well as cell‐scaffold interactions in double and triple cocultures. In addition, the integrity of the scaffolds and their fibrous topography could be visualized by their autofluorescence, mainly excited at 488 nm, although a high intensity sometimes masked the F‐actin staining of the cells. Since the CTG staining decreased with time and cell division, a culture time of 5 days was not exceeded. Images of negative IF staining controls omitting the primary ABs can be found in Figure S4 (Supporting Information).

**Figure 5:**
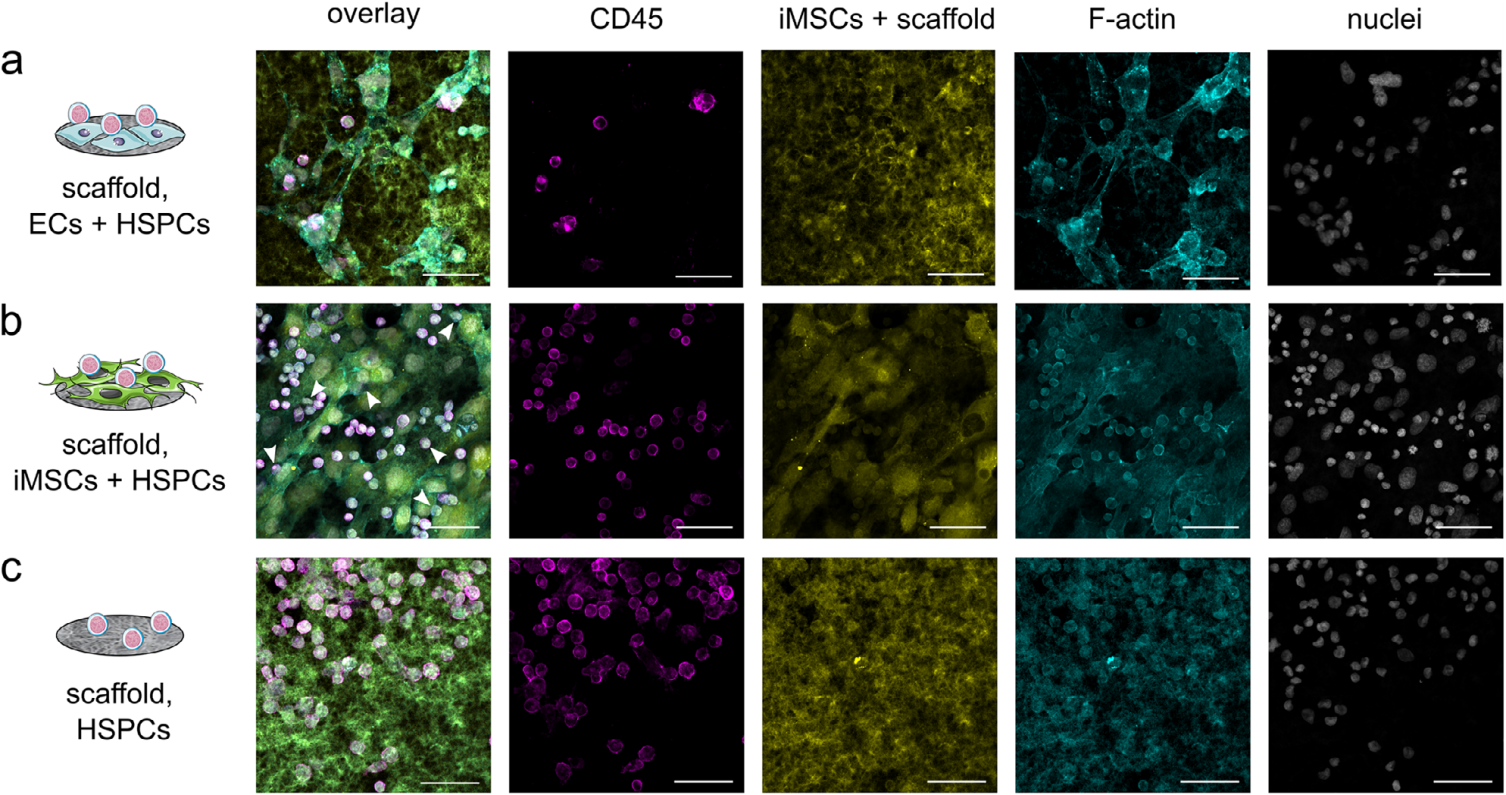
Localization, interaction and morphology of cells cultured on fibrinogen scaffolds. The cells (a: HMEC‐1 cells + HSPCs, b: iMSC#3 cells + HSPCs, c: HSPCs) were cultured for a period of 5 days, subsequently IF stained and analyzed via microscopy. The culture conditions are described on the left. Images of the different channels are depicted in columns. In the first column, overlay images of all channels are given for every culture condition, followed by images of the CD45 staining (pink), iMSC#3 staining and scaffold autofluorescence (yellow), F‐actin staining (turquoise) and nucleus staining (white). White arrowheads in the overlay channel images indicate HSPCs that are located underneath the iMSC#3 cells. Four independent experiments, each containing two technical replicates of every condition, were performed. The images (4 per condition in each experiment) were taken with a confocal laser‐scanning microscope (cLSM) in the 10× objective using the Airyscan detector in the multiplex mode (SR‐4Y). The scale bar represents 50 µm.

In general, similar observations regarding cell localization, morphology, and cell–cell interactions were made when comparing double and triple cocultures on the scaffolds. However, these observations are better visible in the images of the double cocultures (**Figure** **5**) due to the lower number of cell layers. When cultured on the scaffolds, HMEC‐1 cells formed mesh‐like structures and protruded into the fibrous scaffold structure (Figure 5a), aligning with the model's aim to mimic fenestrated endothelium. In contrast, iMSC#3 cells formed a dense cell layer (Figure 5b). HSPCs mainly showed round or slightly polarized morphologies (round with small protrusions) and were positively stained for CD45 (cell periphery, Figure 5c). In cocultures with HMEC‐1 cells, HSPCs were commonly located on top or beside the ECs, indicating a direct interaction. When cocultured with iMSC#3 cells, some of the HSPCs were located beneath the mesenchymal cells (Figure 5b, white arrowheads). In these conditions, HSPCs were also located on the scaffolds and not as close to the cells as in the cocultures with the ECs. HSPCs in monocultures on the scaffolds (Figure 5c) were not homogeneously distributed and often formed cell clusters. As the scaffolds were loosely clamped, small cavities and pits formed in which the cell clusters were located.

Images of the multiple layers of the triple cocultures, captured using z‐stacks, can be found in Figure S5 (Supporting Information). To illustrate the spatial distribution of the cells, 3D reconstruction videos as well as videos containing all z‐layers were created (Videos S1–S5, Supporting Information). Within these z‐stacks, the successful formation of the different cell layers on both sides of the scaffolds was confirmed. Cell localization, morphology, and cell–cell interactions in the triple cultures were similar to the observations made in the double cocultures.

In summary, these results show that the fibrinogen scaffolds were suitable to establish stable cocultures of microvascular EC and MSC layers separated by a nanofibrous, fenestrated membrane structure. HSPCs were introduced from both sides, resembling either their residing in the vascular BM niche or their homing to the niche from the circulation.

### 2.4 HSPC Migration in Vascular Niche Model

BM sinusoids are leaky, discontinuous structures, enabling the egress of HSPCs from the niche into the circulation as well as their homing back to the niche. Consequently, allowing HSPC migration is essential for a suitable basement membrane mimetic of a vascular niche model. For this reason, the spatial distribution of the HSPCs was analyzed to assess their ability to migrate across the nanofibrous scaffold.

Over the 5‐day culture period, HSPCs frequently migrated from the initial seeding layer through the fibrinogen scaffolds, especially in areas of high HSPC density. However, it has to be stated that the majority of HSPCs was not transmigrating. This was an expected result that reflects the physiological behavior of HSPCs in the niche, in which only a small subset of HSPCs respond to local environmental cues and undergo transmigration.^[^ ^68^, ^69^ ^]^ In the current study, migration behavior of HSPCs differed between conditions and also between samples of the same condition. Moreover, it was observed that the scaffolds appear to contain areas of varying thickness, which can be explained by the undulated microtopography described previously.^[^ ^59^, ^70^ ^]^ Due to this topography and the tensile forces of the seeded cells, some scaffolds possessed small holes in locally thin areas, which probably influenced HSPC migration behavior. For this reason, the results of the migration analysis must be interpreted with caution. However, some trends across conditions could be identified. To semi‐quantify observations regarding HSPC migration, images from four independent experiments were analyzed and the position of HPSCs with respect to the different cell layers was assessed. For this purpose, a distinction was made between three categories of HSPC localization: in the top cell layer, underneath the first cell layer and underneath the scaffold. An image was classified as “migration positive”, as soon as one migrated HSPC was found. Scaffolds or areas with holes were excluded from the migration analysis. In the triple cocultures, HSPCs were mostly located underneath the scaffold (69% and 75% of images, **Table** **1**), whereas in mono and double cocultures this only occurred in up to 10% of the images. In the cocultures with iMSC#3 cells, HSPCs often migrated below the iMSC#3 layer, appearing beneath it in 25% of triple coculture images and in over 60% of double coculture images (Table 1). In all other conditions, no migration underneath the first cell layer was found. Interestingly, in double cocultures with HMEC‐1 cells, HSPCs always remained in their seeded layer (100% of images, Table 1).

**Table 1:** Migration of HSPCs in the different experimental conditions after a culture period of 5 days. The scaffolds had a thickness of 2.7 ± 0.4 µm and a highly porous architecture with fiber diameters of approximately 230 nm in the dry state and 340 nm in the wet state as previously described by Suter et al.^[^ ^59^ ^]^ HSPCs were assigned to three categories based on their localization. The number (n_images_) and percentage (%_images_) of images in which HSPCs showed the respective localization is given. Data from four independent experiments is summarized (32 images per experimental condition; in some conditions, less images were taken due to disruptions, holes or wrinkles in the scaffolds). The plus in the condition description indicates that the respective cells were seeded together onto the same side of the scaffold.

Experimental condition	HSPCs remained in top cell layer		HSPCs underneath 1^st^ cell layer		HSPCs underneath scaffold	
	n_images_	%_images_	n_images_	%_images_	n_images_	%_images_
Scaffold, HSPCs	19	90%	n/a	n/a	2	10%
Scaffold, ECs + HSPCs	32	100%	0	0%	0	0%
Scaffold, iMSCs + HSPCs	8	29%	17	61%	3	11%
ECs, scaffold, iMSCs + HSPCs	2	6%	8	25%	22	69%
iMSCs, scaffold, ECs + HSPCs	6	25%	0	0%	18	75%

**Figure 6:**
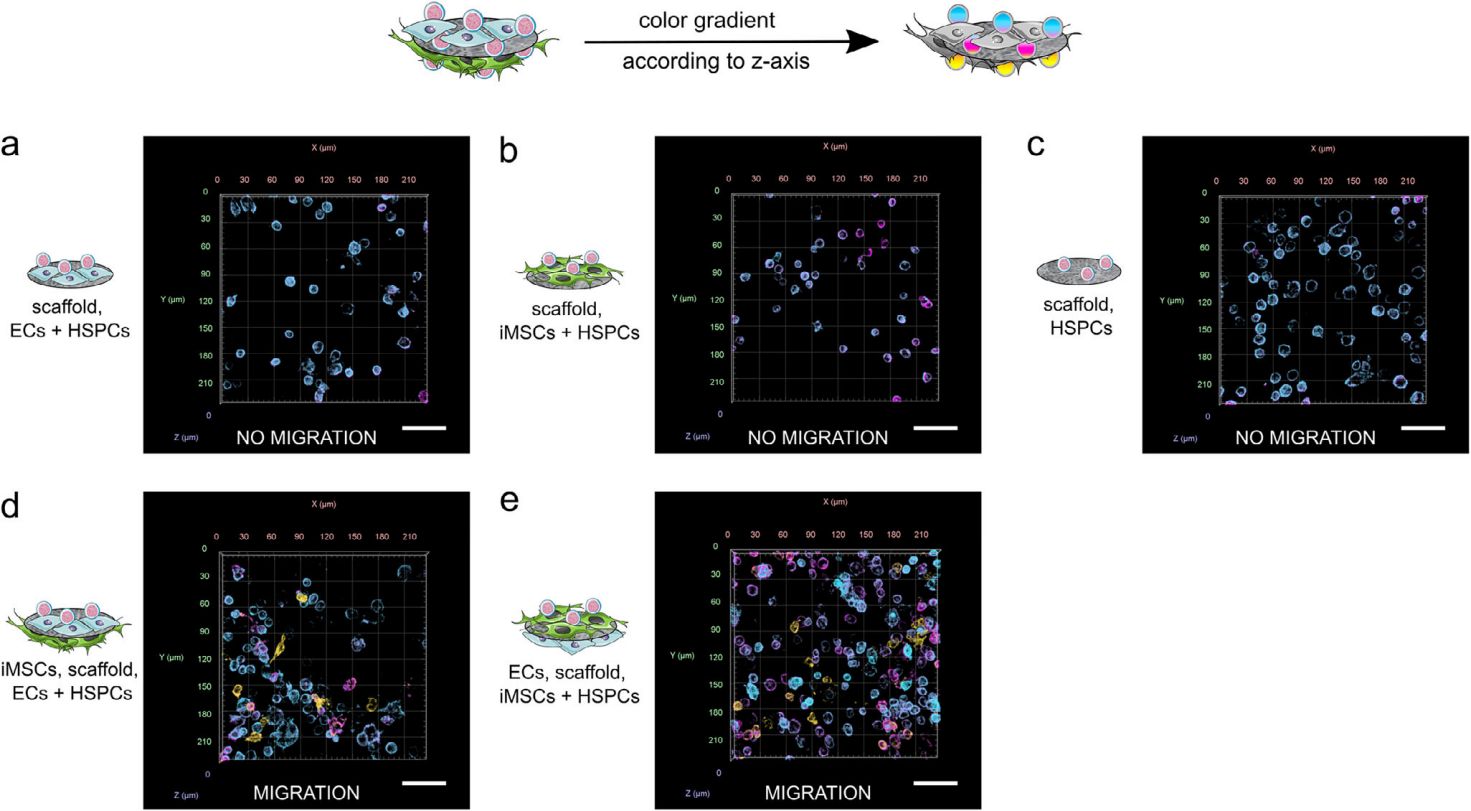
HSPC migration after a culture period of 5 days is illustrated by representative *z*‐axis colored image stacks. HSPCs underneath the scaffold (migration) were observed more frequently in both triple cocultures (d,e) compared to the double cocultures (a,b) and the single culture of HSPCs (c). For this analysis, a color gradient according to the *z*‐axis was applied to representative image stacks of CD45 stained HSPCs. As a result, HSPCs in the same *z*‐layer have the same color. The HSPCs in the top layer can be identified in blue, the HSPCs underneath the scaffold in yellow and HSPCs in intermediate layers in pink. The different experimental conditions (a–e) are described next to the *z*‐stack images. Four independent experiments, each containing two technical replicates of every condition, were performed. For these analyses, 8 *z*‐stacks per condition in each experiment were taken with a confocal laser‐scanning microscope (cLSM) in the 10× objective using the Airyscan detector in the multiplex mode (SR‐4Y). The analysis was performed using arivis. The scale bars represent 50 µm.

In addition to the quantification (Table 1), the migration was visualized by color‐coding the HSPCs within the different layers according to their position along the *z*‐axis (representative images in **Figure** **6**). As with quantification, HSPCs located underneath the scaffold (Figure 6, yellow) were observed more frequently in both triple cocultures (Figure 6, d–e) compared to the other conditions (Figure 6, a–c). In addition, HSPCs were most frequently localized in the intermediate layers (Figure 6, pink) when seeded onto iMSC#3 cells.

Thus, these results indicate that the developed vascular niche model effectively mimicked the discontinuous nature of sinusoidal vessels in the BM, allowing transmigration of HSPCs through the different cell layers as well as through the nanofibrous fibrinogen scaffolds.

### 2.5 HSPC Proliferation and Differentiation in the Vascular Niche Model

**Figure 7:**
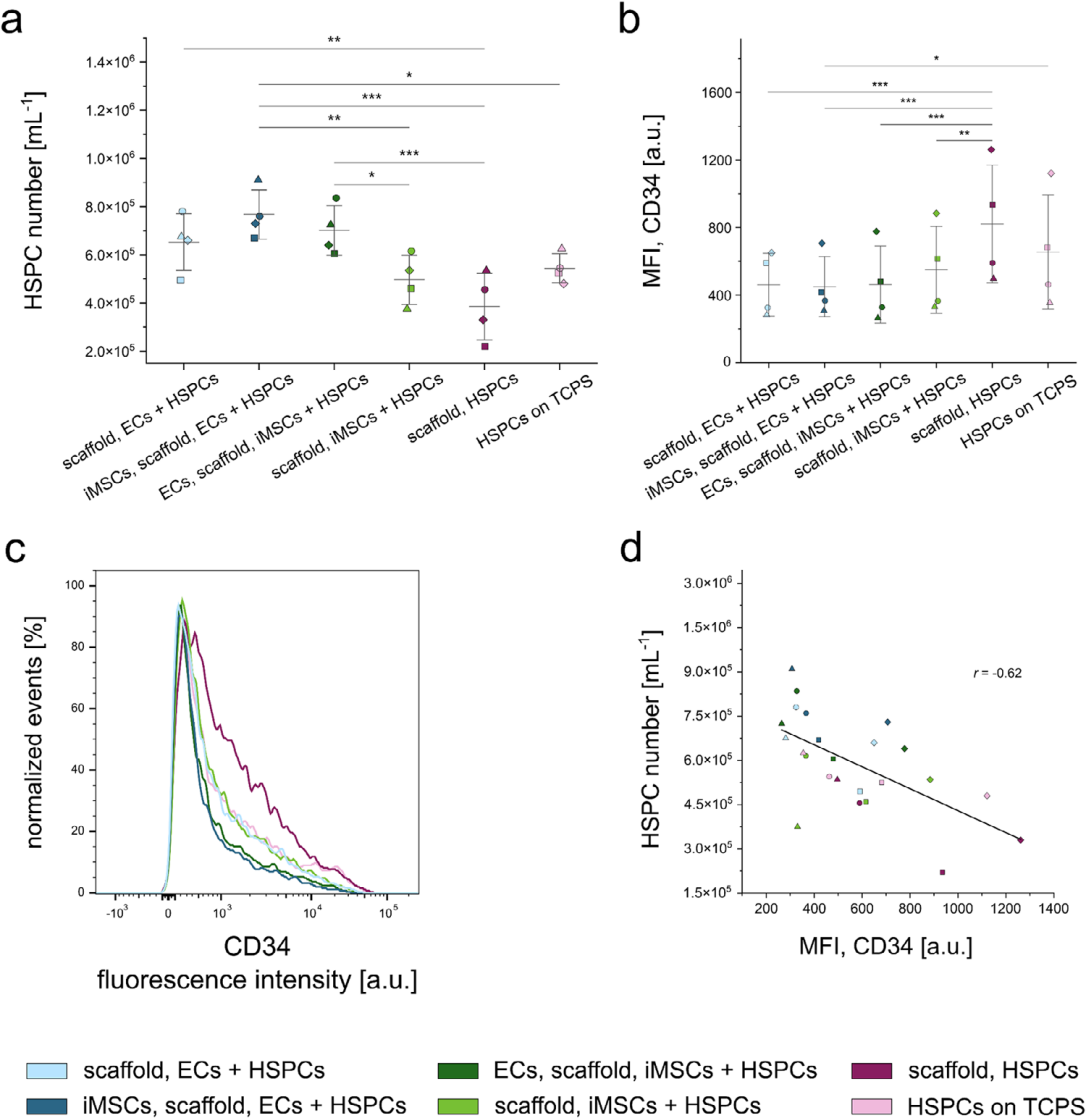
HSPC number and abundance of CD34 after culture on fibrinogen scaffolds. HSPCs were cultured for a culture period of 5 days and subsequently analyzed via flow cytometry. a) HSPC number per mL is shown for the different culture conditions. b) The median fluorescence intensity (MFI) of the CD34 staining is shown for the HSPCs from different culture conditions. c) Histograms show the fluorescence intensity of the CD34 staining of HSPCs cultured in different experimental conditions (representative data of experiment n1). d) The HSPC number per mL is shown in relation to the MFI of the CD34 staining of HSPCs cultured under different experimental conditions. Results of four independent experiments (n1–n4) are shown, indicated by different symbols (n1: square, n2: circle, n3: triangle, n4: diamond), each performed in duplicates. For enumeration and flow cytometry, HSPCs from the technical replicates of the same condition were combined. The culture conditions are illustrated by different colors. Error bars indicate ± SD of mean from four independent experiments. For statistical analysis, a two‐way ANOVA with Tukey post‐hoc test was performed (^*^ *p* ≤ 0.05, ^**^ *p* ≤ 0.01, ^***^ *p* ≤ 0.001).

Natural niches not only regulate HSPC adhesion and migration, but also proliferation and differentiation. Therefore, HSPC proliferation was assessed by cell enumeration, and differentiation by analyzing the abundance of the surface marker CD34, whose expression decreases when HSPCs differentiate toward mature lineages.^[^ ^71^, ^72^ ^]^ After 5 days of culture, nonadherent HSPCs were rinsed from the fibrinogen scaffolds, counted, and analyzed via flow cytometry. HSPCs exhibited highly increased cell numbers across all conditions compared to the initial seeding number of 10 000 cells per condition (**Figure** **7**a), but high donor variability resulted in large standard deviations (SDs) between experimental runs. The highest HSPC numbers were observed in both triple cocultures (increase of 47‐fold, Table S1, Supporting Information), whereas HSPC monocultures on the scaffolds showed the lowest counts (Figure 7a) (increase of 29‐fold, Table S1, Supporting Information). HSPCs cultured alone on fibrinogen scaffolds possessed significantly more CD34 compared to the other conditions, except for HSPCs on TCPS (Figure 7b). The lowest CD34 abundance was observed for HSPCs cultured only with HMEC‐1 cells as well as for HSPCs from the triple cocultures. In the double coculture with iMSC#3 cells, the abundance of CD34 in HSPCs was slightly higher. These trends are consistent with the histograms in Figure 7c (representative data of n1). Notably, CD34 histogram curves were more similar in both triple cocultures as well as in both double cocultures compared to the other curves. Figure 7d illustrates a negative correlation (Pearson coefficient *r* = −0.62) between the cell number per mL and the median fluorescence intensity (MFI) of the CD34 staining, suggesting that increased cell numbers are associated with decreased CD34 abundance.

These results show that the fibrinogen scaffolds as well as the different cell types influence the proliferation and differentiation of HSPCs, emphasizing the importance of considering the basement membrane and using relevant cell types in the development of vascular niche models. Furthermore, the data illustrate the potential of the different cell types to communicate with each other, even across cell layers and the nanofibrous fibrinogen scaffold.

### 2.6 Cytokine Production

**Figure 8:**
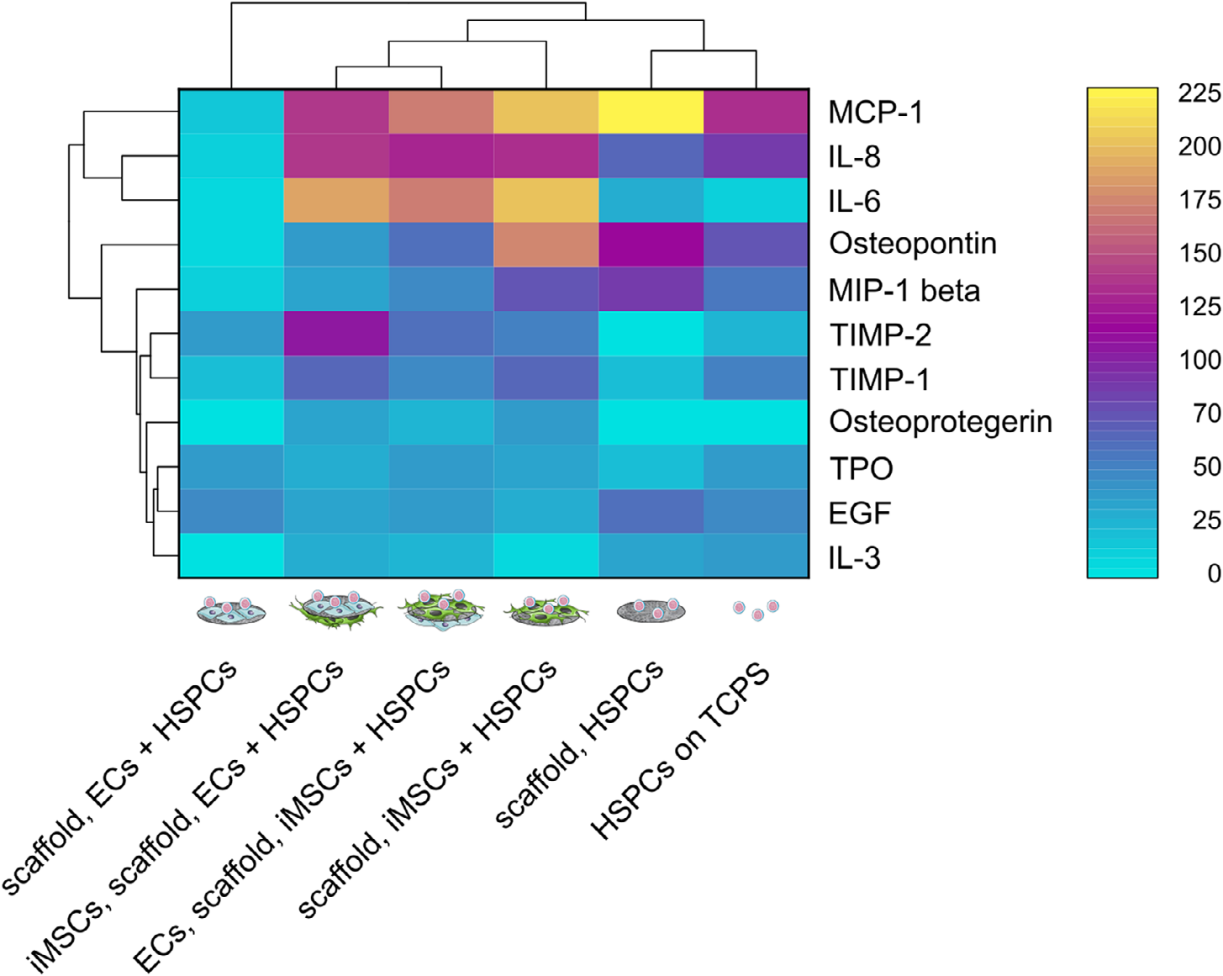
Cytokine production differed between the experimental conditions. A membrane‐based cytokine array was used to analyze medium supernatants after the culture of HSPCs, HMEC‐1 and iMSC#3 cells for a period of 5 days. Supernatants of four independent experiments were pooled for the array. The biclustered heatmap displays all cytokines that exceeded a threshold of 25%. The dendrogram at the top shows the hierarchical relationship between the experimental conditions.

Results of the migration and proliferation studies indicated that the cells within the vascular niche model communicated not only via direct cell‐cell contacts but also indirectly via soluble factors. Therefore, the medium supernatants from the different culture conditions were screened for 80 cytokines using a membrane‐based array. Of those, 11 cytokines were considered as regulated since they exceeded a threshold of 25%, which was set to separate signal from noise (Figure S6, Supporting Information). These cytokines were visualized in a biclustered heatmap (**Figure** **8**). The hierarchical dendrogram reveals the similarity between the experimental conditions with regard to the detected cytokines by grouping more similar conditions into one cluster. It can be seen that all experimental conditions are grouped in one cluster, with the exception of the double coculture of HSPCs and HMEC‐1 cells. In addition, both HSPC monocultures as well as all iMSC#3‐containing conditions are grouped into further clusters, indicating their similarity regarding secreted and consumed cytokines. Besides, both triple cocultures showed greater similarity to each other than to the iMSC#3 and HSPC double coculture, as shown by an additional cluster. The clustering indicates that the cell types present in the respective conditions have the greatest influence on the secreted and consumed cytokines in the medium supernatants. Cultures containing iMSC#3 cells generally exhibited the highest cytokine levels and substantially altered the cytokine composition of the supernatants. In these cultures, monocyte chemoattractant protein 1 (MCP‐1), interleukin 8 (IL‐8), and IL‐6 were highly abundant, particularly in the iMSC#3 and HSPC double coculture. In triple cocultures, cytokine concentrations were mostly similar, except for elevated levels of tissue inhibitor of metalloproteinase 2 (TIMP‐2) when HSPCs were seeded onto HMEC‐1 cells. Osteopontin was predominantly present in the iMSC#3 and HSPC double coculture, along with slightly higher TIMP‐1 and macrophage inflammatory protein 1 beta (MIP‐1 beta) levels compared to other conditions. When comparing both HSPC monocultures, the effect of the fibrinogen scaffolds on the cytokine composition of the medium supernatants can be estimated. The supernatant of HSPCs cultured on scaffolds contained elevated levels of MCP‐1, osteopontin, and MIP‐1 beta as well as moderate levels of IL‐8 and epidermal growth factor (EGF) compared to the supernatant of HSPCs cultured on TCPS. Interestingly, the fewest cytokines were found in the supernatant of the double coculture of HMEC‐1 and HSPCs, with only low EGF, TPO, and TIMP‐2 levels detected.

In summary, the presented results show a clear influence of the cell type composition of the vascular niche model on the cytokine profile, reflecting the observed differences in cell migration, proliferation, and differentiation.

## 3 Discussion

Vascular niches are home to the majority of HSPCs in the BM.^[^ ^5^, ^12^ ^]^ The sinusoidal vascular niche does not only regulate self‐renewal and differentiation like the other niche entities, but is also the primary place of HSPC trafficking.^[^ ^13^ ^]^ This process involves egress from the niche as observed with circadian rhythms in homeostasis^[^ ^73^ ^]^ and at accelerated rates during stress‐induced recruitment in the course of infections or clinical mobilization for HSPC transplantation.^[^ ^74^ ^]^ In addition to egress, HSPCs home to the sinusoidal niche from the circulation after homeostatic trafficking or HSPC transplantation.^[^ ^74^, ^75^ ^]^ To understand and investigate these processes in a human cell setting, mimicking the different layers that need to be traversed during transendothelial migration across sinusoidal vessels is essential. Previous in vitro models of the vascular BM niche relied on methods established to mimic larger vessel structures, thereby neglecting the intricate fenestrated morphology of BM sinusoids characterized by microvascular ECs and a discontinuous thin basement membrane. The aim of the current study was therefore to replicate these delicate structures of the sinusoidal vascular niches and to assess their functionality in terms of HSPC support and trafficking.

The nanofibrous fibrinogen scaffolds used to resemble the basement membrane, were the central element of the current in vitro model. To our knowledge, previously developed models of the vascular niche did not explicitly include a basement membrane mimetic. In some BM‐on‐a‐chip models, porous membranes made from synthetic materials such as polydimethylsiloxane (PDMS)^[^ ^34^ ^]^ or polyethylene terephthalate (PET)^[^ ^76^ ^]^ were used to separate endosteal and vascular niche compartments. However, the use of a natural biomaterial like fibrinogen offers several advantages. Cells can directly adhere to fibrinogen via integrin binding toward two Arginine‐Glycine‐Aspartate (RGD) sites,^[^ ^57^, ^77^, ^78^ ^]^ which make it a favorable material for cell culture applications. When using synthetic materials, additional cell adhesive coatings or surface modifications are needed.^[^ ^79^, ^80^ ^]^ Another limitation of using synthetic materials as basement membrane mimetic is that these materials cannot be remodeled by cells. The basement membrane in vivo is known to be a dynamic structure, which can be reorganized or altered through protein synthesis or degradation.^[^ ^81^ ^]^ Fibrinogen is involved in forming the provisional matrix in early stages of wound healing in vivo and can be replaced by other ECM proteins in later phases.^[^ ^82^, ^83^ ^]^ Using a natural material such as fibrinogen, which is highly biodegradable,^[^ ^57^ ^]^ allows for a more accurate mimicry of the basement membrane. In addition, thin fibrinogen scaffolds with the required properties were reproducibly obtained due to its controllable self‐assembling properties.^[^ ^55^, ^58^, ^59^ ^]^ Moreover, self‐assembled fibrinogen nanofibers were already found to degrade in the presence of different enzymes^[^ ^65^ ^]^ and supported the adhesion of blood platelets^[^ ^70^ ^]^ as well as cocultures of human fibroblasts and keratinocytes,^[^ ^84^ ^]^ which make them ideal candidates to mimic the basement membrane in the current in vitro model. The used scaffolds had a thickness of 2.7 ± 0.4 µm, a high roughness (≈120 nm) and a highly porous architecture with fiber diameters of approximately 230 nm in the dry state and 340 nm in the rehydrated state as described previously by Suter et al.^[^ ^59^ ^]^ This resembles the fibrous network structure of the basement membrane in vivo, which contains fiber diameters in the range of 30 to 400 nm as well as pores that allow the diffusion of small solutes and transmigration of cells.^[^ ^47^ ^]^ Only the thickness of the basement membrane in vivo is reported to be much thinner (50–300 nm).^[^ ^47^ ^]^ However, the used fibrinogen scaffolds can still be evaluated as appropriate to mimic the basement membrane, as thinner membranes would be too fragile to handle in cell culture experiments. In comparison, commercially available cell culture membranes out of synthetic materials (e.g., transwells) are much thicker and range from 10 to 50 µm.

The selection of a suitable material, such as nanofibrous fibrinogen scaffolds, is only one part of constructing a realistic in vitro model. Equally important is the choice of appropriate cell types that can accurately represent the in vivo environment. In the vascular niche, MSCs and ECs are the most crucial supporter cell types of HSPCs.^[^ ^9^, ^26^, ^85^, ^86^, ^87^ ^]^ To account for the stromal cells, iMSC#3 cells, which have been shown to exhibit the behavior and characteristics of BM MSCs,^[^ ^88^ ^]^ were used in the current in vitro model. They origin from BM MSCs, which were immortalized via retroviral transduction of telomerase reverse transcriptase.^[^ ^88^ ^]^ Since these cells possess a more stable phenotype compared to primary BM MSCs, the use of iMSC#3 cells was beneficial to simplify the developed in vitro model and to avoid donor‐to‐donor variability. In addition, HMEC‐1 cells were used to mimic the fenestrated single‐layer of microvascular ECs that lines sinusoidal blood vessels in vivo.^[^ ^22^ ^]^ Throughout biomaterial research, HUVECs are the most commonly used ECs for various different applications due to their ease of access and culture.^[^ ^89^ ^]^ Accordingly, most of the in vitro models of the vascular niche developed so far also used HUVECs.^[^ ^33^, ^34^, ^64^, ^76^, ^90^, ^91^, ^92^, ^93^ ^]^ However, since these cells are macrovascular ECs obtained from the umbilical vein, they are not optimal for use in every in vitro model, as ECs from different organs and tissues possess a high heterogeneity.^[^ ^94^, ^95^ ^]^ In particular, differences between macro‐ and microvascular ECs have been described regarding phenotype, marker expression, and function.^[^ ^96^, ^97^, ^98^ ^]^ Thus, the use of microvascular HMEC‐1 cells allows to replicate the in vivo situation in BM sinusoids more closely and is therefore advantageous compared to the use of HUVECs. By seeding the fibrinogen scaffolds with iMSC#3 cells from one side and HMEC‐1 cells from the other side, a suitable material was combined with appropriate cell types to accurately mimic the microenvironment of the vascular niche.

In order to obtain a functional in vitro model, not only the choice of material and cell type is important, but also how these cell types adhere to the material and interact with each other. Especially when resembling fragile structures such as BM sinusoids, cell‐cell and cell‐matrix adhesions are crucial to ensure the cohesion of the assembled structures. Furthermore, cell adhesion influences HSPC behavior and is known to be a crucial process in HSPC‐niche‐interactions,^[^ ^91^, ^100^ ^]^ which is reflected in the morphology of adhered cells. In the current study, adherent HSPCs could be observed in all conditions. Most of the HSPCs exhibited round or slightly polarized morphologies (round with small protrusions) and formed clusters when cultured on the nanofibrous scaffolds. This morphology and cluster formation was also observed by Chua et al., who cultured HSPCs on electrospun nanofibers.^[^ ^101^, ^102^ ^]^ HSPCs also showed round or slightly polarized morphologies when cocultured with iMSC#3 and/or HMEC‐1 cells. This is in line with previous studies also reporting that the majority of HSPCs showed a round morphology when adhering to MSCs.^[^ ^103^ ^]^ When an elongated, highly polarized morphology of HSPCs in close contact with adjacent niche cells was observed, the studies were performed on smooth surfaces and in the presence of cytokines such as, stromal cell‐derived factor 1 (SDF‐1), known to enhance migration of HSPCs.^[^ ^103^, ^104^, ^105^ ^]^ Thus, the observed HSPC morphology in the current study was as expected. Unlike the HSPCs, which maintained consistent morphology across all experimental conditions, HMEC‐1 and iMSC#3 cells exhibited noticeable differences in adhesion to the nanofibrous fibrinogen scaffolds and the FN‐coated glass coverslips. Both cell types displayed less defined stress fibers, smaller cell spreading areas and more protrusions on the nanofibrous scaffolds compared to the FN‐coated glass surface. This observed morphology might be attributed to differences between the two molecules such as molecular weight (fibrinogen: 340 kDa, FN: 440 kDa),^[^ ^106^, ^107^ ^]^ structure (fibrinogen: trimer of dimers, FN: dimer),^[^ ^106^, ^107^ ^]^ surface charge,^[^ ^108^, ^109^ ^]^ cell adhesion sites (fibrinogen: RGD and Alanine‐Glycine‐Aspartic acid (AGD) motifs,^[^ ^110^ ^]^ FN: RGD in the type III FN domain 10, synergy side in type III FN domain 9)^[^ ^111^ ^]^ and/or biological function.^[^ ^112^, ^113^, ^114^ ^]^ Furthermore, the porous nanofiber networks and the flat FN‐coated glass differ largely in topography and roughness, which are known to affect cell adhesion similarly to the observed differences in the adhesion behavior of HMEC‐1 and iMSC#3.^[^ ^115^, ^116^, ^117^ ^]^ Specifically, previous studies found a less pronounced actin cytoskeleton, smaller cell spreading areas and more protrusions in fibroblasts and keratinocytes on nanofibrous fibrinogen scaffolds in comparison to planar fibrinogen.^[^ ^59^, ^84^ ^]^ In addition, the observed HMEC‐1 and iMSC#3 morphology aligns with cell morphologies of fibroblasts, epithelial cells, MSCs or ECs on other nanofibrous materials described previously.^[^ ^54^, ^118^, ^119^, ^120^ ^]^ Moreover, less defined vinculin patterns and fewer vinculin spots were observed after adhesion of iMSC#3 and HMEC‐1 cells to the fibrinogen scaffold compared to adhesion to FN‐coated glass. In line with these observations, other studies also described more diffuse vinculin staining and lower vinculin expression in cells cultured on nanofibers compared to cells cultured on flat surfaces.^[^ ^118^, ^119^, ^120^ ^]^ Taken together, the observed differences for HMEC‐1 and iMSC#3 cells grown on both surfaces can mainly be attributed to the fibrous surface structure and undulated topography of the scaffolds in comparison to the coated glass. In addition, tight interaction of both cell types with the fibrinogen scaffolds could be observed. This indicates the suitability of the material for the vascular niche model, as it effectively ensured cohesion between stromal and endothelial components while allowing HSPC trafficking.

The migration and trafficking of HSPCs within the different cell layers and the basement membrane mimetic was particularly evident in both triple cocultures, although the majority of the cells did not migrate. One explanation for the enhanced migration in the triple cultures compared to the other culture conditions could be the secretion of inflammatory cytokines by the cells involved, as inflammation in vivo induces the migration of HSPCs from the BM into the circulation.^[^ ^121^, ^122^, ^123^, ^124^ ^]^ Besides, mobilized HSPCs are attracted by chemotactic cytokines to sides of inflammation or injured tissue.^[^ ^125^ ^]^ In the current study, supernatants obtained from cocultures of HSPCs and iMSC#3 cells contained high amounts of MCP‐1, IL‐8, and IL‐6, which were shown to exhibit proinflammatory properties.^[^ ^126^, ^127^, ^128^ ^]^ Similar observations were made by Aggarwal et al., who found that the coculture with PBMCs significantly increased the secretion of IL‐8 and IL‐6 by BM MSCs.^[^ ^129^ ^]^ In addition, the fibrinogen scaffolds could have even enhanced the secretion of these cytokines as this effect of fibrinogen has also been demonstrated in BM MSCs and in mice.^[^ ^130^, ^131^ ^]^ Along with these inflammatory cytokines, high concentrations of osteopontin, MCP‐1 and MIP‐1 beta were found in the supernatants of the double coculture of HSPCs and iMSC#3 cells. Osteopontin was probably produced by myeloid progenitors, as it was shown that monocytes upregulated endogenous osteopontin production according to serum levels of inflammatory markers.^[^ ^132^ ^]^ In addition, increased osteopontin concentrations were shown to elevate the production of MCP‐1 and MIP‐1 beta.^[^ ^132^, ^133^ ^]^ Taken together, these findings indicate that the fibrinogen scaffolds and the coculture with HSPCs enhanced the production of pro‐inflammatory cytokines by iMSC#3 cells. These cytokines probably upregulated endogenous osteopontin in myeloid progenitors, which in turn increased the concentrations of MCP‐1 and MIP‐1 beta. Besides their inflammatory character, chemotactic properties were reported for some cytokines found in the supernatants. IL‐8 was shown to enhance HSPC migration^[^ ^128^, ^134^ ^]^ and MCP‐1 as well as MIP‐1 beta enhanced the migration of monocytes.^[^ ^132^, ^133^ ^]^ Overall, the high concentrations of inflammatory and chemotactic cytokines are in line with the observed HSPC migration, particularly in the triple cocultures.

Additionally, decreased scaffold integrity due to degradation may have facilitated HSPC migration, particularly in the triple cocultures. ECs and MSCs express metalloproteinases (MMPs)^[^ ^135^, ^136^ ^]^ capable to degrade ECM molecules such as fibrinogen.^[^ ^137^, ^138^, ^139^ ^]^ The increased overall cell number in the triple cocultures and the cell seeding from both sides of the scaffolds could have elevated scaffold degradation, as was previously observed in the presence of plasmin and a combination of urokinase and plasminogen.^[^ ^65^ ^]^ Supporting this hypothesis, high concentrations of TIMP‐1 and TIMP‐2, inhibitors of MMPs,^[^ ^140^, ^141^ ^]^ were found in supernatants of conditions where iMSC#3 cells were present. The concentration of TIMPs was especially high in triple cocultures, where HSPCs were seeded onto HMEC‐1 cells. Accordingly, also the most HSPC migration was found in this condition. High TIMP levels may indicate a matrix‐protective reaction of the iMSC#3 cells toward increased scaffold degradation, as it was shown that MSCs secrete TIMPs to protect the niche from MMP‐induced disruption.^[^ ^142^ ^]^

In general, when comparing all experimental conditions regarding the cytokines detected in the supernatants, most cytokines were secreted by the iMSC#3 cells. This was an expected result, as MSCs are known to secrete a variety of soluble factors and cytokines to regulate or support other cell types.^[^ ^143^, ^144^, ^145^ ^]^ Interestingly, the overall fewest number of cytokines was found in the supernatant of the double coculture of HSPCs and HMEC‐1 cells. This result might indicate that the HMEC‐1 cells consume a high number of cytokines, particularly MCP‐1, osteopontin and MIP‐1 beta, since higher concentrations of these cytokines were detected in the single culture of HSPCs on the scaffolds. Supporting this hypothesis, it can be seen that the concentration of the three cytokines is lower in the triple cocultures compared to the double coculture of HSPCs and iMSC#3 cells, where the HMEC‐1 cells were absent.

Another interesting observation was that the localization of the HSPCs in relation to the other cell types differed between HMEC‐1/HSPC cocultures and iMSC#3/HSPC cocultures. In the latter, HSPCs predominantly resided beneath iMSC#3 cells, corroborating literature on HSPC and MSC interactions.^[^ ^146^, ^147^, ^148^, ^149^ ^]^ Since this behavior of HSPCs is a commonly described phenomenon, the compartment between the scaffold and stromal cells might provide a specialized niche in which the HSPCs preferred to reside. In contrast to iMSC#3/HSPC cocultures, HSPCs in HMEC‐1/HSPC cocultures consistently remained on top of the ECs or besides them. In the case of migrated HSPCs in these conditions, the HSPCs migrated completely through the scaffold, not settling between ECs and scaffold. This behavior reflects the in vivo situation, in which HSPCs transmigrate across blood vessels and EC layers rather than residing between ECs and their associated basement membrane, to which the ECs are tightly connected via adhesion.^[^ ^13^, ^150^, ^151^ ^]^

Overall, the differences in HSPC localization indicate that the triple cocultures on nanofibrous fibrinogen scaffolds accurately resemble the trafficking dynamics in the natural vascular BM niche.

Beyond allowing the trafficking of HSPCs, a crucial aspect of an accurate vascular niche model is its ability to regulate HSPC proliferation and differentiation.^[^ ^93^, ^152^ ^]^ The overall observed high proliferation of HSPCs in the current study was expected due to the supplemented cytokines (TPO, SCF, Flt‐3 ligand, IL‐3) known to support proliferation, survival and expansion of HSPCs.^[^ ^153^, ^154^, ^155^, ^156^ ^]^ The observed high variability in HSPC numbers can be attributed to donor‐to‐donor variances, which are commonly reported in HSPC studies, as CD34^+^ cell populations are highly heterogeneous.^[^ ^157^, ^158^ ^]^ In general, higher CD34 abundance correlated with lower HSPC numbers. It was expected that the HSPCs proliferate and differentiate toward more mature progeny over the culture period of 5 days and, therefore, lose CD34 to a certain extent.^[^ ^71^, ^72^ ^]^ Concordantly, Wang et al.,^[^ ^159^ ^]^ for example, reported that the percentage of CD34^+^ cells decreased to 32% after culturing HSPCs for 7 days in a culture medium similar to the one used in the current study. Therefore, HSPCs at various stages of differentiation, but no fully mature cells were expected. CD34 was used in the current study to estimate the extent of differentiation and to compare the influence of the fibrinogen scaffold as well as the influence of the different cell types on HSPC differentiation. The lowest HSPC numbers along with the highest CD34 abundance were found in the monoculture on scaffolds. Therefore, the absence of other cell types and the direct interaction of the HSPCs with the scaffold appears to promote the maintenance of primitive progenitors. In addition, the nanofibrous topography of the fibrinogen scaffolds could have enhanced HSPC expansion, as several other studies also observed high numbers of CD34^+^ HSPCs after culture on nanofibers.^[^ ^160^ ^]^ Furthermore, nanofibers can be referred to as intermediate between 2D and 3D due to their high surface area‐to‐volume ratio and it is known that a 3D environment promotes the maintenance of primitive progenitors.^[^ ^31^ ^]^ Besides, the loose clamping of the scaffolds led to the formation of small cavities and pits, which could have further intensified the spatial effect of the undulated microtopography.^[^ ^59^, ^70^ ^]^ In accordance with the low HSPC numbers found in this condition, high levels of osteopontin, a cytokine which has been shown to suppress the proliferation of HSPCs,^[^ ^161^, ^162^ ^]^ were found in the respective media supernatants. In addition, as discussed before, higher concentrations of osteopontin were shown to elevate the production of MCP‐1 and MIP‐1 beta,^[^ ^132^, ^133^ ^]^ which could also be an explanation for the higher cytokine concentrations in the HSPC single culture on the scaffold compared to the culture on TCPS. The highest HSPC numbers along with low CD34 abundance were found in both triple cocultures. Consistent with this finding, a low concentration of osteopontin was found in the supernatants of these conditions. In addition, high concentrations of the cytokines TIMP‐1 and IL‐8, which were both shown to enhance HSPC proliferation,^[^ ^134^, ^163^ ^]^ were found in the supernatants of the triple cocultures. A high proliferation of HSPCs and a loss of CD34, as seen in these conditions, indicates an enhanced differentiation toward mature progenitors.^[^ ^71^, ^72^ ^]^ These results are consistent with other in vitro studies that have also demonstrated the ability of ECs and MSCs to promote HSC proliferation and differentiation.^[^ ^90^, ^149^, ^164^ ^]^ Previous studies have also shown that more primitive HSPCs preferentially adhere to the niche and to niche cells such as MSCs than mature HSPCs.^[^ ^146^, ^149^ ^]^ Therefore, it is possible that the adherent HSPCs that were not harvested with the culture supernatant, were more primitive than the nonadherent cell fraction analyzed by flow cytometry.

Taken together, the results indicate that the developed vascular niche model allowed both, HSPC proliferation and differentiation, with clear impact of the basement membrane mimetic and the supporting niche cells, similar to natural niche environment.

## 4 Conclusion

In this study, a suitable in vitro model of the vascular HSC niche was successfully developed using self‐assembled fibrinogen nanofibers and three different cell types of the BM microenvironment. Using microvascular ECs, a thin and fenestrated endothelium was created, which is characteristically found in BM sinusoids in vivo. Besides, iMSC#3 cells were included in the model as a second supporter cell type for the HSPCs to account for the stromal compartment of the vascular niche. Within the study, suitable coculture conditions for the three different cell types were successfully established. Self‐assembled fibrinogen nanofiber scaffolds proved to be a suitable basement membrane mimetic due to their structure, cytocompatibility, and cell adhesive properties. The use of microvascular ECs and the fact that the model allowed the transmigration of HSPCs, is an advantage that exceeds the capabilities of previously developed models. In particular, a custom‐made clamping system enabled the seeding of different cell types onto both sides of the scaffold, which could also be used for scaffolds of different materials or the cocultivation of other cell types. Given the promising characteristics of the developed vascular niche model, future studies could involve tailoring it even closer to the natural archetype. This could be achieved by using scaffolds made from a combination of ECM proteins such as FN, laminin and collagen IV, which have been shown to be part of the BM basement membrane. Incorporating additional cell types and repeating the experiments under hypoxia could also be beneficial, as low oxygen tension is an important factor influencing cell behavior in the vascular niche. Moreover, the model is well‐suited for the use in vascular‐niche‐ or BM‐on‐chip models. In conclusion, we present a versatile technology for mimicking basement membranes in tissue engineering of delicate, discontinuous blood vessels as found in BM vascular niches and other tissues.

## 5 Experimental Section

### Materials/Chemicals and Reagents

All ABs for flow cytometry (Table S2, Supporting Information), Viobility Dye 405/520, MACS Compbeads (Anti‐REA), MACS CD34 MicroBead Kit (Human) and MACS MS Columns were purchased from Miltenyi Biotec (Bergisch Gladbach, Germany). ABs and dyes for IF staining (Table S3, Supporting Information) were obtained from Thermo Fisher Scientific (Waltham, MA, USA); Phalloidin‐iFluor 555 Reagent from abcam (Cambridge, USA). Dulbecco′s phosphate buffered saline (DPBS) without MgCl_2_ and CaCl_2_ (DPBS (−/−)), DPBS with MgCl_2_ and CaCl_2_ (DPBS (+/+)), Fetal Bovine Serum (FBS), Normal Goat Serum (NGS), Bovine Serum Albumin (BSA), Penicillin‐Streptomycin (P/S), paraformaldehyde (PFA), and FN were purchased from Merck (Darmstadt, Germany).

### Cell Lines and Cell Culture

The human microvascular endothelial cell line 1 (HMEC‐1; CRL‐3243, RRID: CVCL_0307) was purchased from ATCC (LGC Standards GmbH, Wesel, Germany) in passage 22. HMEC‐1 cells were cultured in MCDB 131 Medium (PAN Biotech, Aidenbach, Germany) containing 10% FBS, 10 × 10^−3^ m ROTI Cell Glutamine solution (Carl Roth, Karlsruhe, Germany), 10 ng Epidermal Growth Factor (human, recombinant (*E. coli*), PromoCell, Heidelberg, Germany) and 1 µg mL^−1^ Hydrocortisone (Merck). HMEC‐1 cells were used in passages 30‐34. The immortalized human BM mesenchymal cell line (iMSC#3) (RRID: CVCL_B5PE) was kindly provided by Prof. Dr. Ola Myklebost (University of Bergen, Department of Clinical Science) in passage 42. iMSC#3 cells were cultured in Alpha MEM Eagle medium (PAN Biotech, Aidenbach, Germany) containing 10% FBS and 2 µg mL^−1^ Puromycin dihydrochloride (Merck). iMSC#3 cells were used in passages 54‐71.

### HSPC Isolation from Cord Blood

CD34^+^ HSPCs were isolated from human UCB. The UCB samples were kindly provided by Prof. Dr. med. Constantin von Kaisenberg (Hannover Medical School, Department for Obstetrics, Gynecology and Reproductive Medicine, Research Obstetric Biobank). The withdrawal of the UCB was approved by the ethics committee of the Hannover Medical School (ethic vote no. 1303‐2012) and performed after information and written declaration of consent from the donors. UCB samples were excluded when the donor was suffering from infectious diseases or diseases that need permanent medical treatment and only accepted if the birth took place after the 35^th^ week of pregnancy. All performed studies were approved by the central ethics committee of the Leibniz University Hannover (EV LUH 06/2019). The peripheral blood mononuclear cells were separated from granulocytes and erythrocytes via density gradient centrifugation using Pancoll (density: 1.077 g mL^−1^, PAN Biotech, Aidenbach, Germany) and separation tubes (Leucosep, Greiner Bio‐One, Frickenhausen, Germany). Remaining erythrocytes were eliminated using an Erylysis buffer. CD34^+^ cells were isolated via magnetic activated cell sorting (MACS) and only samples with a purity ≥ 95% (flow cytometry) were used for experiments. 1 × 10^5^ cells mL^−1^ were expanded for 48 h in Hematopoietic Progenitor Expansion Medium XF (PromoCell) containing 1% Cytokine Mix E (PromoCell) and 1% P/S before seeding for experiments.

### Fibrinogen Scaffold Fabrication

Nanofibrous fibrinogen scaffolds were prepared by salt‐induced self‐assembly.^[^ ^55^, ^65^ ^]^ In brief, the scaffolds were fabricated by drying a purified 2.5 mg mL^−1^ fibrinogen solution (100% clottable, Merck) in the presence of 2.5× phosphate‐buffered saline (pH 7.4, Thermo Fisher Scientific) on cleaned glass coverslips with a diameter of 18 mm. Cross‐linking of the fibers was performed using formaldehyde vapor (37% formaldehyde, 1 µL cm^−3^ incubation volume). For fabrication of the immobilized scaffolds, the coverslips were functionalized with 5% (3‐aminopropyl)triethoxysilane (APTES, Sigma, Steinheim, Germany) prior to nanofiber assembly. To obtain free‐standing scaffolds for cocultivation with cells on both sides, fibrinogen nanofibers were detached from glass slides by washing with deionized water.

### Cell Culture on Clamped Free‐standing Fibrinogen Scaffolds

The experiments were performed according to the workflow depictured in Figure 4. Sterilized fibrinogen scaffolds (UV light, 30 min) were detached from glass coverslips and the free‐standing scaffolds were clamped using PVC rings, screws and nuts. The detailed detachment and clamping process is described in the Supporting Information and shown in Figure S7 (Supporting Information) as well as in Video S6 (Supporting Information).

For cell seeding, silicone rings (two‐component duplicating silicone (Replisil 22 N A+B, Siltecs, Ulm, Germany) were placed onto the PVC rings. The cells were seeded sequentially (cells per scaffold: HMEC‐1: 150 000, iMSC#3: 10 000, HSPCs: 5 000). After seeding one cell type, the clamped scaffolds were incubated for 3 h at 37 °C and 5% CO_2_ to ensure cell adhesion before seeding the next cell type. The iMSC#3 cells were stained with a CMFDA dye (5‐chloromethylfluorescein diacetate; CellTracker Green, CTG, 10 × 10^−6^ m) before seeding to label and localize the iMSC#3 cells in double and triple cocultures. Since the CTG staining decreases with time and cell division, a culture time of 5 days was not exceeded, during which unambiguous detection of the labeled cells was ensured (Figure S4, Supporting Information). The detailed seeding procedure of all cell types is described in the Supporting Information and depicted in Figure S8 (Supporting Information) as well as in Video S7 (Supporting Information). In every experiment, six experimental conditions were compared: two double cocultures (scaffold, ECs + HSPCs and scaffold, iMSCs + HSPCs), two triple cocultures (iMSCs, scaffold, ECs + HSPCs and ECs, scaffold, iMSCs + HSPCs), and two HSPC monoculture controls (HSPCs on scaffold and HSPCs on TCPS). The plus indicates that two cell types were cultured on the same side of the fibrinogen scaffold. The cells were cultured on the fibrinogen scaffolds for 5 days. All media used in the experiments contained 1% P/S and the iMSC#3 medium was used without Puromycin dihydrochlorid. The composition of the media used for double and triple cocultures can be found in Table S4 (Supporting Information).

### Flow Cytometry Analysis

HSPCs were analyzed for their percentage of CD34 directly after isolation from UCB and before seeding onto the scaffolds to ensure sufficient purity. In addition, HSPCs were analyzed after 5 days of culture on the scaffolds for the abundance of CD34 and CD45. The clamped scaffolds were transferred into DPBS (+/+)‐filled 6 well plates with the HSPC‐seeded side facing the well bottom. The nonadherent HSPCs were rinsed from the scaffolds by gently swirling the plate. In this way, the scaffolds were washed twice. The culture medium and the DBPS from the washing steps were combined for each condition and centrifuged to collect the nonadherent HSPCs. 20 000–50 000 HSPCs of every condition were washed twice with Running Buffer (2 × 10^−3^ m EDTA solution (1%) + 0.05% (v/v) BSA in DPBS (−/−)) and stained with the ABs or the respective isotype ABs (10 min, 4 °C). In addition, the cells were stained with a viability dye (15 min, RT). All ABs and dyes used for flow cytometry and the respective dilution are listed in Table S2 (Supporting Information). To detect spillover and correct for it in the analyses, compensation beads (MACS Comp Beads Kit, anti‐REA) were used. To compensate for the spillover of the viability dye, VioGreen labeled ABs were used for the bead staining. All steps containing fluorophore‐coupled dyes were performed in the dark. Running Buffer was always used cold (4 °C). The samples were measured using the FACSVerse from BD Biosciences (East Rutherford, NJ, USA) with three lasers (405, 488, and 640 nm). The obtained data was analyzed using FlowJo (v10.9.0). Gating was performed equally in all samples as shown in Figure S10 (Supporting Information).

### IF Staining

After removal of the nonadherent HSPCs for flow cytometry analysis, the cell‐grown scaffolds were subjected to IF staining. All steps were performed in 6‐well plates under gentle rotation using an orbital shaker and the scaffolds remained clamped during the whole procedure. ABs, dyes and the used dilutions are listed in Table S3 (Supporting Information). In the first step, the cell‐grown scaffolds were fixed using a 4% PFA solution (15 min, RT). After three washing steps, the cells were permeabilized for 10 min with Triton X‐100 (0.1% solution in ddH_2_O, Merck), washed again (3×) and blocked for 1 h with NGS (10% in DPBS (+/+)). Subsequently, the samples were washed (3×) and incubated with the primary ABs. Staining controls omitting primary ABs were incubated with the blocking solution (10% NGS in DPBS (+/+)). Then, the samples were washed (3×) and incubated for 1 h with the secondary AB mixture, which also contained phalloidin and DAPI, followed by an extensive washing step (3 × 10 min). After the staining procedure, the fibrinogen scaffolds were unclamped and mounted on glass slides with Mowiol (24% (w/v) Glycerol (Merck), 9.6% (w/v) Mowiol 4‐88 (Merck), 2.5% (w/v) Dabco 33‐LV (Merck), 0.1 m TRIS‐HCl (pH 8.5) (Merck) in ddH_2_O). The detailed unclamping and mounting process is described in the Supporting Information and shown in Figure S9 (Supporting Information) as well as in Video S8 (Supporting Information).

### Microscopy and Image Analysis

The IF‐stained samples were analyzed using a confocal laser‐scanning microscope (cLSM, AxioObserver 7, LSM 980, Airyscan detector) with six lasers (405, 445, 488, 514, 561, and 639 nm). For image analysis, ZEN (3.3) and arivis Pro (4.0) from Zeiss were used.

### Analysis of Cell Spreading Area and Cell Solidity

Using images of single cultures, the cell spreading area and the cell solidity of HSPCs, HMEC‐1, and iMSC#3 cells on FN‐coated glass and immobilized fibrinogen scaffolds were measured by analyzing the F‐actin staining via Fiji. First, the image type was changed to RGB color, followed by adjusting the brightness of the color threshold. In a next step the “wand tracing tool” was used to mark the outlines of the cells. When cells were overlapping, the “pencil tool” was used for separation. Subsequently, the cell outlines were added to the ROI manager and both parameters were measured.

### Migration Analysis

Microscopy image stacks of IF‐stained samples were analyzed regarding HSPC localization. Three categories were assigned: HSPCs in top layer, HSPCs underneath 1^st^ cell layer, and HSPCs underneath scaffold. The number and percentage of images in which HSPCs showed the respective localization was enumerated. To visualize HSPC migration behavior, a color gradient was applied to the image stacks according to the *z*‐axis of the 647 nm channel, which showed the CD45‐stained HSPCs. As a result, HSPCs in the same *z*‐layer appeared in the same color (see Figure 6).

### Experiments with Immobilized Fibrinogen Scaffolds

Cells cultured on immobilized fibrinogen scaffolds and FN‐coated glass coverslips were used for vinculin staining and SEM analysis. The coverslips were heat sterilized (180 °C, 3 h), coated with a FN solution (20 µg mL^−1^ in sterile ddH_2_O) for 1 h at 37 °C and 5% CO_2_ and washed with sterile ddH_2_O. The immobilized scaffolds were sterilized (UV, 30 min) before cell seeding. HMEC‐1 and iMSC#3 cells (5 000 per scaffold/coverslip) were seeded in their growth medium. After 3 h, the medium was exchanged to TCM and HSPCs were added. After 4 days of culture, medium was removed and the samples were washed twice with pre‐warmed DPBS (+/+). Vinculin staining was performed according to the described IF procedure. In a last step, the cell‐covered scaffolds and coverslips were mounted on glass slides with Mowiol or fixed and dehydrated for SEM analysis.

### SEM Analysis

For SEM analysis, cells were fixed (20 min, 4% PFA) and rinsed in DPBS (+/+) (2 × 5 min) and ddH_2_O (3×5 min). Subsequently, the samples were gradually dehydrated by incubation in increasing concentrations of EtOH in ddH_2_O (50% EtOH for 2.5 h; 75% EtOH overnight at 4 °C; 87.5% EtOH for 15 min; 93.8% EtOH for 15 min; 96.9% EtOH for 15 min; 98.4% EtOH for 15 min) and air‐dried overnight. The dehydrated samples were mounted on aluminum stubs using conductive carbon tabs, sputtered with gold (1 min, Sputter Coater S150A, Edwards, Irvine, CA, USA) and analyzed in a Quanta 200 SEM (FEI, Dreieich, Germany; SE detector; WD 9 – 10 mm). Operation voltage differed according to the substrate: 15 kV for FN‐coated glass, 8 kV for fibrinogen scaffolds.

### Cytokine Analysis

After 5 days of culture, medium supernatants were collected and stored at −20 °C until all experimental runs were completed. The Human Cytokine Antibody Array C5 (RayBiotech, Norcross, GA, USA) was performed according to the manufacturers’ instructions. All steps were performed under gentle rotation (orbital shaker) to ensure even sample distribution. For each condition and for the medium control (medium cultured without cells), one membrane was incubated with 1 mL of the respective supernatant (combination from all experimental runs) overnight at 4 °C. Chemiluminescence was detected using an UVP ChemStudio PLUS touch (Analytic Jena, Jena, Germany; binning: 2×2, exposure: 15 sec). Spot intensities were obtained from inverted images using the “Protein Analyzer Tool” of ImageJ (Fiji^[^ ^165^ ^]^). For further analysis, the value of the internal negative control of each membrane was subtracted and obtained negative values were set to zero. In a next step, all values were normalized according to the respective internal positive control of the membrane. Subsequently, the signal of the medium control was subtracted. From these obtained values, only the ones that exceeded a threshold of 25%, were considered as regulated cytokines. To define this threshold, values of all cytokines were displayed in a diagram and a cut‐off was set to discriminate background noise from signals clearly exceeding this noise (Figure S6, Supporting Information). The heatmap showing the remaining 11 cytokines was created using OriginPro (2023, 10.0.0.154).

### Statistical Analysis and Preparation of Figures

OriginPro was used for statistical analyses and the generation of diagrams. If not stated otherwise, data is presented as mean ± standard deviation (SD). To test for significances, a two‐way ANOVA followed by a Tukey post hoc test was performed (^*^ *p* ≤ 0.05, ^**^ *p* ≤ 0.01, ^***^ *p* ≤ 0.005). For the analysis of the cell spreading area and the cell solidity, a *t*‐test was performed (^*^ *p* ≤ 0.05, ^**^ *p* ≤ 0.01, ^***^ *p* ≤ 0.005). Figures were prepared using Inkscape (1.0.2‐2; graphs, microscopy images, presentation of data) and MS PowerPoint (workflow, schematics; using Servier Medical Art, provided by Servier, licensed under a Creative Commons Attribution 3.0 unported license).

### Ethics approval statement

The withdrawal of the UCB was approved by the ethics committee of the Hannover Medical School (ethic vote no. 1303‐2012) and performed after information and written declaration of consent from the donors. All performed studies were approved by the central ethics committee of the Leibniz University Hannover (EV LUH 06/2019).

## Conflict of Interest

The authors declare no conflict of interests.

## Supporting information



Supporting Information: Supporting figures, tables, methods, description of supporting videos.

Supplemental Video 1: 3D reconstruction video and z‐stack of condition "scaffold, ECs + HSPCs"

Supplemental Video 2: 3D reconstruction video and z‐stack of condition "iMSCs, scaffold, ECs + HSPCs"

Supplemental Video 3: 3D reconstruction video and z‐stack of condition "ECs, scaffold, iMSCs + HSPCs"

Supplemental Video 4: 3D reconstruction video and z‐stack of condition "scaffold, iMSCs + HSPCs"

Supplemental Video 5: 3D reconstruction video and z‐stack of condition "scaffold, HSPCs"

Supplemental Video 6: Detachment and clamping of fibrinogen scaffolds

Supplemental Video 7: Seeding of cells on clamped fibrinogen scaffolds

Supplemental Video 8: Unclamping and mounting of fibrinogen scaffolds

## Data Availability

The data that support the findings of this study are available from the corresponding author upon reasonable request.

## References

[1] S. Doulatov , F. Notta , E. Laurenti , J. E. Dick , Cell Stem Cell 2012, 10, 120. 22305562 10.1016/j.stem.2012.01.006

[2] M. Kasbekar , C. A. Mitchell , M. A. Proven , E. Passegué , Cell Stem Cell 2023, 30, 1403. 37865087 10.1016/j.stem.2023.09.013 PMC10842631

[3] G. Ferrari , A. J. Thrasher , A. Aiuti , Nat. Rev. Genet. 2021, 22, 216. 33303992 10.1038/s41576-020-00298-5

[4] M. A. Rieger , T. Schroeder , Cold Spring Harbor Perspect. Biol. 2012, 4, a008250. 10.1101/cshperspect.a008250 PMC3504436 23209149

[5] G. M. Crane , E. Jeffery , S. J. Morrison , Nat. Rev. Immunol. 2017, 17, 573. 28604734 10.1038/nri.2017.53

[6] D. Lucas , Curr. Opin. Hematol. 2021, 28, 36. 33177411 10.1097/MOH.0000000000000621 PMC7769132

[7] R. Schofield , Blood Cells 1978, 4, 7. 747780

[8] S. J. Morrison , D. T. Scadden , Nature 2014, 505, 327. 24429631 10.1038/nature12984 PMC4514480

[9] S. Pinho , P. S. Frenette , Nat. Rev. Mol. Cell Biol. 2019, 20, 303. 30745579 10.1038/s41580-019-0103-9 PMC6483843

[10] I. Beerman , T. C. Luis , S. Singbrant , C. Lo Celso , S. Méndez‐Ferrer , Exp. Hematol. 2017, 50, 22. 28189651 10.1016/j.exphem.2017.01.008 PMC5466495

[11] K. D. Kokkaliaris , L. Kunz , N. Cabezas‐Wallscheid , C. Christodoulou , S. Renders , F. Camargo , A. Trumpp , D. T. Scadden , T. Schroeder , Blood 2020, 136, 2296. 32766876 10.1182/blood.2020006574 PMC8209553

[12] M. Acar , K. S. Kocherlakota , M. M. Murphy , J. G. Peyer , H. Oguro , C. N. Inra , C. Jaiyeola , Z. Zhao , K. Luby‐Phelps , S. J. Morrison , Nature 2015, 526, 126. 26416744 10.1038/nature15250 PMC4850557

[13] T. Itkin , S. Gur‐Cohen , J. A. Spencer , A. Schajnovitz , S. K. Ramasamy , A. P. Kusumbe , G. Ledergor , Y. Jung , I. Milo , M. G. Poulos , A. Kalinkovich , A. Ludin , O. Kollet , G. Shakhar , J. M. Butler , S. Rafii , R. H. Adams , D. T. Scadden , C. P. Lin , T. Lapidot , Nature 2016, 532, 323. 27074509 10.1038/nature17624 PMC6450701

[14] U. Harjes , C. Verfaillie , P. Carmeliet , Dev. Cell 2016, 37, 210. 27165553 10.1016/j.devcel.2016.04.016

[15] Y. Xiao , C. S. McGuinness , W. S. Doherty‐Boyd , M. Salmeron‐Sanchez , H. Donnelly , M. J. Dalby , Biomaterials 2022, 286, 121568. 35580474 10.1016/j.biomaterials.2022.121568

[16] Z. Yang , R. Dong , X. Mao , X. C. He , L. Li , Curr. Opin. Cell Biol. 2024, 86, 102284. 37995509 10.1016/j.ceb.2023.102284

[17] Y. Kunisaki , I. Bruns , C. Scheiermann , J. Ahmed , S. Pinho , D. Zhang , T. Mizoguchi , Q. Wei , D. Lucas , K. Ito , J. C. Mar , A. Bergman , P. S. Frenette , Nature 2013, 502, 637. 24107994 10.1038/nature12612 PMC3821873

[18] Y. Kunisaki , P. S. Frenette , Int. J. Hematol. 2014, 99, 699. 24756874 10.1007/s12185-014-1580-4

[19] S. Comazzetto , B. Shen , S. J. Morrison , Dev. Cell 2021, 56, 1848. 34146467 10.1016/j.devcel.2021.05.018 PMC8282762

[20] F. Matteini , M. A. Mulaw , M. C. Florian , Front. Immunol. 2021, 12, 738204. 34858399 10.3389/fimmu.2021.738204 PMC8631970

[21] A. L. Pereira , S. Galli , C. Nombela‐Arrieta , HemaSphere 2024, 8, 133. 10.1002/hem3.133 PMC11289431 39086665

[22] M. Hassanshahi , A. Hassanshahi , S. Khabbazi , Y.‐W. Su , C. J. Xian , Angiogenesis 2017, 20, 427. 28956197 10.1007/s10456-017-9577-2

[23] C. I. Weidner , T. Walenda , Q. Lin , M. M. Wölfler , B. Denecke , I. G. Costa , M. Zenke , W. Wagner , Sci. Rep. 2013, 3, 3372. 24284763 10.1038/srep03372 PMC3842544

[24] S. Kumar , H. Geiger , Trends Mol. Med. 2017, 23, 799. 28801069 10.1016/j.molmed.2017.07.003 PMC5600322

[25] W. S. Doherty‐Boyd , H. Donnelly , M. P. Tsimbouri , M. J. Dalby , Exp. Hematol. 2024, 135, 104232. 38729553 10.1016/j.exphem.2024.104232

[26] M. de Lima , I. McNiece , S. N. Robinson , M. Munsell , M. Eapen , M. Horowitz , A. Alousi , R. Saliba , J. D. McMannis , I. Kaur , P. Kebriaei , S. Parmar , U. Popat , C. Hosing , R. Champlin , C. Bollard , J. J. Molldrem , R. B. Jones , Y. Nieto , B. S. Andersson , N. Shah , B. Oran , L. J. N. Cooper , L. Worth , M. H. Qazilbash , M. Korbling , G. Rondon , S. Ciurea , D. Bosque , I. Maewal , et al., N. Engl. J. Med. 2012, 367, 2305. 23234514 10.1056/NEJMoa1207285 PMC3805360

[27] R. S. Mehta , R. M. Saliba , K. Cao , I. Kaur , K. Rezvani , J. Chen , A. Olson , S. Parmar , N. Shah , D. Marin , A. Alousi , C. Hosing , U. Popat , P. Kebriaei , R. Champlin , M. de Lima , D. Skerrett , E. Burke , E. J. Shpall , B. Oran , Biol. Blood Marrow Transplant. 2017, 23, 1359. 28506845 10.1016/j.bbmt.2017.05.002 PMC6453532

[28] Q. Qu , L. Liu , G. Chen , Y. Xu , X. Wu , D. Wu , Cytotherapy 2016, 18, 452. 26857234 10.1016/j.jcyt.2015.12.005

[29] M. Bruschi , T. Vanzolini , N. Sahu , A. Balduini , M. Magnani , A. Fraternale , Front. Bioeng. Biotechnol. 2022, 10, 968086. 36061428 10.3389/fbioe.2022.968086 PMC9428512

[30] G. Born , M. Nikolova , A. Scherberich , B. Treutlein , A. García‐García , I. Martin , J. Tissue Eng. 2021, 12, 20417314211044855. 34616539 10.1177/20417314211044855 PMC8488506

[31] C. Chatterjee , P. Schertl , M. Frommer , A. Ludwig‐Husemann , A. Mohra , N. Dilger , T. Naolou , S. Meermeyer , T. C. Bergmann , A. A. Calleja , C. Lee‐Thedieck , Acta Biomater. 2021, 132, 129. 33813090 10.1016/j.actbio.2021.03.061

[32] A. Mansoorifar , R. Gordon , R. Bergan , L. E. Bertassoni , Adv. Funct. Mater. 2021, 31, 2006796. 35422682 10.1002/adfm.202006796 PMC9007546

[33] M. R. Nelson , D. Ghoshal , J. C. Mejías , D. F. Rubio , E. Keith , K. Roy , Biomaterials 2021, 270, 120683. 33556648 10.1016/j.biomaterials.2021.120683

[34] D. B. Chou , V. Frismantas , Y. Milton , R. David , P. Pop‐Damkov , D. Ferguson , A. MacDonald , Ö. V. Bölükbaşı , C. E. Joyce , L. S. Moreira Teixeira , A. Rech , A. Jiang , E. Calamari , S. Jalili‐Firoozinezhad , B. A. Furlong , L. R. O'Sullivan , C. F. Ng , Y. Choe , S. Marquez , K. C. Myers , O. K. Weinberg , R. P. Hasserjian , R. Novak , O. Levy , R. Prantil‐Baun , C. D. Novina , A. Shimamura , L. Ewart , D. E. Ingber , Nat. Biomed. Eng. 2020, 4, 394. 31988457 10.1038/s41551-019-0495-z PMC7160021

[35] P. E. Bourgine , Trends Biotechnol. 2025. 10.1016/j.tibtech.2025.05.028 40541487

[36] S. Frenz‐Wiessner , S. D. Fairley , M. Buser , I. Goek , K. Salewskij , G. Jonsson , D. Illig , B. zu Putlitz , D. Petersheim , Y. Li , P.‐H. Chen , M. Kalauz , R. Conca , M. Sterr , J. Geuder , Y. Mizoguchi , R. T. A. Megens , M. I. Linder , D. Kotlarz , M. Rudelius , J. M. Penninger , C. Marr , C. Klein , Nat. Methods 2024, 21, 868. 38374263 10.1038/s41592-024-02172-2 PMC11093744

[37] D. E. Glaser , M. B. Curtis , P. A. Sariano , Z. A. Rollins , B. S. Shergill , A. Anand , A. M. Deely , V. S. Shirure , L. Anderson , J. M. Lowen , N. R. Ng , K. Weilbaecher , D. C. Link , S. C. George , Biomaterials 2022, 280, 121245. 34810038 10.1016/j.biomaterials.2021.121245 PMC10658812

[38] T. Heike , T. Nakahata , Biochim. Biophys. Acta Mol. Cell Res. 1592, 2002, 313. 10.1016/s0167-4889(02)00324-5 12421675

[39] Y. K. Bozhilov , I. Hsu , E. J. Brown , A. C. Wilkinson , Cells 2023, 12, 896. 36980237 10.3390/cells12060896 PMC10046976

[40] B. Çelebi , D. Mantovani , N. Pineault , Biomed. Mater. 2011, 6, 55011. 10.1088/1748-6041/6/5/055011 21931196

[41] I. Leisten , R. Kramann , M. S. Ventura Ferreira , M. Bovi , S. Neuss , P. Ziegler , W. Wagner , R. Knüchel , R. K. Schneider , Biomaterials 2012, 33, 1736. 22136713 10.1016/j.biomaterials.2011.11.034

[42] K. G. DeFrates , R. Moore , J. Borgesi , G. Lin , T. Mulderig , V. Beachley , X. Hu , Nanomaterials 2018, 8, 457. 29932123 10.3390/nano8070457 PMC6071022

[43] K. S. Ogueri , C. T. Laurencin , ACS Nano 2020, 14, 9347. 32678581 10.1021/acsnano.0c03981 PMC7484273

[44] S. Nemati , S. Kim , Y. M. Shin , H. Shin , Nano Convergence 2019, 6, 36. 31701255 10.1186/s40580-019-0209-y PMC6838281

[45] A. Hiwrale , S. Bharati , P. Pingale , A. Rajput , Heliyon 2023, 9, 18917. 10.1016/j.heliyon.2023.e18917 PMC10477438 37674834

[46] R. N. Palchesko , Y. Sun , L. Zhang , J. M. Szymanski , Q. Jallerat , A. W. Feinberg , Springer Handbook of Nanomaterials, Springer, Berlin Heidelberg 2013, p. 977.

[47] P. Jain , S. B. Rauer , M. Möller , S. Singh , Biomacromolecules 2022, 23, 3081. 35839343 10.1021/acs.biomac.2c00402 PMC9364315

[48] G. E. Wnek , M. E. Carr , D. G. Simpson , G. L. Bowlin , Nano Lett. 2003, 3, 213.

[49] D. Gugutkov , J. Gustavsson , M. P. Ginebra , G. Altankov , Biomater. Sci. 2013, 1, 1065. 32481872 10.1039/c3bm60124b

[50] M. J. Mirzaei‐Parsa , H. Ghanbari , N. Bahrami , S. Hadadi‐Abianeh , R. Faridi‐Majidi , J. Polym. Eng. 2018, 38, 945.

[51] I. Alghoraibi , S. Alomari , Handbook of Nanofibers, Springer, Cham 2019, pp. 1–46.

[52] C. J. Bosques , B. Imperiali , J. Am. Chem. Soc. 2003, 125, 7530. 12812489 10.1021/ja035360b

[53] T. Koga , K. Kitamura , N. Higashi , Chem. Commun. 2006, 4897. 10.1039/b611679e 17136239

[54] N. Suter , S. Stebel , C. Rianna , M. Radmacher , D. Brüggemann , Biofabrication 2020, 13, 015007. 33135668 10.1088/1758-5090/abb744

[55] K. Stapelfeldt , S. Stamboroski , P. Mednikova , D. Brüggemann , Biofabrication 2019, 11, 025010. 30829217 10.1088/1758-5090/ab0681

[56] T. Li , X.‐M. Lu , M.‐R. Zhang , K. Hu , Z. Li , Bioact. Mater. 2022, 11, 268. 34977431 10.1016/j.bioactmat.2021.09.029 PMC8668426

[57] T. Rajangam , S. S. A. An , Int. J. Nanomed. 2013, 8, 3641. 10.2147/IJN.S43945 PMC3792008 24106425

[58] K. Stapelfeldt , S. Stamboroski , I. Walter , N. Suter , T. Kowalik , M. Michaelis , D. Brüggemann , Nano Lett. 2019, 19, 6554. 31418579 10.1021/acs.nanolett.9b02798

[59] N. Suter , A. Joshi , T. Wunsch , N. Graupner , K. Stapelfeldt , M. Radmacher , J. Müssig , D. Brüggemann , Mat. Sci. Eng., C 2021, 126, 112156. 10.1016/j.msec.2021.112156 34082961

[60] M. V. J. Braham , A. S. P. Li Yim , J. Garcia Mateos , M. C. Minnema , W. J. A. Dhert , F. C. Öner , C. Robin , J. Alblas , Adv. Health Mater. 2019, 8, 1801444. 10.1002/adhm.201801444 30941927

[61] C. A. Di Buduo , P.‐A. Laurent , C. Zaninetti , L. Lordier , P. M. Soprano , A. Ntai , S. Barozzi , A. La Spada , I. Biunno , H. Raslova , J. B. Bussel , D. L. Kaplan , C. L. Balduini , A. Pecci , A. Balduini , eLife 2021, 10, 58775. 10.7554/eLife.58775 PMC8169123 34059198

[62] A. O. Khan , A. Rodriguez‐Romera , J. S. Reyat , A.‐A. Olijnik , M. Colombo , G. Wang , W. X. Wen , N. Sousos , L. C. Murphy , B. Grygielska , G. Perrella , C. B. Mahony , R. E. Ling , N. E. Elliott , C. S. Karali , A. P. Stone , S. Kemble , E. A. Cutler , A. K. Fielding , A. P. Croft , D. Bassett , G. Poologasundarampillai , A. Roy , S. Gooding , J. Rayes , K. R. Machlus , B. Psaila , Cancer Discovery 2023, 13, 364. 36351055 10.1158/2159-8290.CD-22-0199 PMC9900323

[63] N. Tamaoki , S. Siebert , T. Maeda , N.‐H. Ha , M. L. Good , Y. Huang , S. K. Vodnala , J. J. Haro‐Mora , N. Uchida , J. F. Tisdale , C. L. Sweeney , U. Choi , J. Brault , S. Koontz , H. L. Malech , Y. Yamazaki , R. Isonaka , D. S. Goldstein , M. Kimura , T. Takebe , J. Zou , D. F. Stroncek , P. G. Robey , M. J. Kruhlak , N. P. Restifo , R. Vizcardo , Cell Rep. Methods 2023, 3, 100460. 37159663 10.1016/j.crmeth.2023.100460 PMC10163025

[64] A. Georgescu , J. H. Oved , J. H. Galarraga , T. Cantrell , S. Mehta , B. M. Dulmovits , T. S. Olson , P. Fattahi , A. Wang , P. L. Candarlioglu , A. Muvaffak , M. M. Kim , S. A. Aydin , J. Seo , E. S. Diffenderfer , A. Lynch , G. S. Worthen , D. D. Huh , Cell Stem Cell 2024, 31, 1847.e6. 39642865 10.1016/j.stem.2024.11.003 PMC11651161

[65] T. Strunk , A. Joshi , M. Moeinkhah , T. Renzelmann , L. Dierker , D. Grotheer , N. Graupner , J. Müssig , D. Brüggemann , ACS Appl. Bio Mater. 2024, 7, 6186. 10.1021/acsabm.4c00761 PMC11409215 39226515

[66] J. L. Bays , K. A. DeMali , Cell. Mol. Life Sci. 2017, 74, 2999. 28401269 10.1007/s00018-017-2511-3 PMC5501900

[67] T. Izard , D. T. Brown , J. Biol. Chem. 2016, 291, 2548. 26728462 10.1074/jbc.R115.686493 PMC4742724

[68] P. Quaranta , L. Basso‐Ricci , R. J. Hernandez , G. Pacini , M. M. Naldini , M. Barcella , L. Seffin , G. Pais , G. Spinozzi , F. Benedicenti , C. Pietrasanta , J. G. Cheong , A. Ronchi , L. Pugni , F. Dionisio , I. Monti , S. Giannelli , S. Darin , F. Fraschetta , G. Barera , F. Ferrua , V. Calbi , M. Ometti , R. Di Micco , F. Mosca , S. Z. Josefowicz , E. Montini , A. Calabria , M. E. Bernardo , M. P. Cicalese , et al., Blood 2024, 143, 1937. 38446574 10.1182/blood.2023022666 PMC11106755

[69] I. B. Mazo , S. Massberg , U. H. von Andrian , Trends Immunol. 2011, 32, 493. 21802990 10.1016/j.it.2011.06.011 PMC3185129

[70] M. Kenny , S. Stamboroski , R. Taher , D. Brüggemann , I. Schoen , Adv. Health Mater. 2022, 11, 2200249. 10.1002/adhm.202200249 PMC11469041 35526111

[71] C. Caux , C. Favre , S. Saeland , V. Duvert , P. Mannoni , I. Durand , J. P. Aubry , J. E. de Vries , Blood 1989, 74, 1287. 2475184

[72] S. Salati , R. Zini , E. Bianchi , A. Testa , F. Mavilio , R. Manfredini , S. Ferrari , Stem Cells 2008, 26, 950. 18192237 10.1634/stemcells.2007-0597

[73] K. Golan , A. Kumari , O. Kollet , E. Khatib‐Massalha , M. D. Subramaniam , Z. S. Ferreira , F. Avemaria , S. Rzeszotek , A. García‐García , S. Xie , E. Flores‐Figueroa , S. Gur‐Cohen , T. Itkin , A. Ludin‐Tal , H. Massalha , B. Bernshtein , A. K. Ciechanowicz , A. Brandis , T. Mehlman , S. Bhattacharya , M. Bertagna , H. Cheng , E. Petrovich‐Kopitman , T. Janus , N. Kaushansky , T. Cheng , I. Sagi , M. Z. Ratajczak , S. Méndez‐Ferrer , J. E. Dick , et al., Cell Stem Cell 2018, 23, 572.e7. 30174297 10.1016/j.stem.2018.08.002

[74] K. Lapid , C. Glait‐Santar , S. Gur‐Cohen , J. Canaani , O. Kollet , T. Lapidot , StemBook, Egress and Mobilization of Hematopoietic Stem and Progenitor Cells: A Dynamic Multi‐facet Process, Vitana ‐ LOEL Nederland BV, Cambridge, MA 2008. 23658994

[75] A. Kumari , K. Golan , E. Khatib‐Massalha , O. Kollet , T. Lapidot , Hematopoietic Stem Cell Niche, Elsevier Science, Saint Louis 2017, p. 85.

[76] C. Sui , J. Zilberberg , W. Lee , Sci. Rep. 2022, 12, 1439. 35087109 10.1038/s41598-022-05520-4 PMC8795452

[77] K. Suehiro , J. Gailit , E. F. Plow , J. Biol. Chem. 1997, 272, 5360. 9030612 10.1074/jbc.272.8.5360

[78] A. Salsmann , E. Schaffner‐Reckinger , F. Kabile , S. Plançon , N. Kieffer , J. Biol. Chem. 2005, 280, 33610. 15955823 10.1074/jbc.M500146200

[79] N. Huettner , T. R. Dargaville , A. Forget , Trends Biotechnol. 2018, 36, 372. 29422411 10.1016/j.tibtech.2018.01.008

[80] M. B. Rahmany , M. van Dyke , Acta Biomater. 2013, 9, 5431. 23178862 10.1016/j.actbio.2012.11.019

[81] N. Khalilgharibi , Y. Mao , Open Biol. 2021, 11, 200360. 33593159 10.1098/rsob.200360 PMC8061686

[82] R. A. Clark , J. M. Lanigan , P. DellaPelle , E. Manseau , H. F. Dvorak , R. B. Colvin , J. Invest. Dermatol. 1982, 79, 264. 6752288 10.1111/1523-1747.ep12500075

[83] P. Olczyk , Ł. Mencner , K. Komosinska‐Vassev , BioMed. Res. Int. 2014, 2014, 747584. 24772435 10.1155/2014/747584 PMC3977088

[84] A. Joshi , T. Nuntapramote , D. Brüggemann , ACS Omega 2023, 8, 8650. 36910955 10.1021/acsomega.2c07896 PMC9996769

[85] J. M. Butler , D. J. Nolan , E. L. Vertes , B. Varnum‐Finney , H. Kobayashi , A. T. Hooper , M. Seandel , K. Shido , I. A. White , M. Kobayashi , L. Witte , C. May , C. Shawber , Y. Kimura , J. Kitajewski , Z. Rosenwaks , I. D. Bernstein , S. Rafii , Cell Stem Cell 2010, 6, 251. 20207228 10.1016/j.stem.2010.02.001 PMC2866527

[86] I. G. Winkler , V. Barbier , B. Nowlan , R. N. Jacobsen , C. E. Forristal , J. T. Patton , J. L. Magnani , J.‐P. Lévesque , Nat. Med. 2012, 18, 1651. 23086476 10.1038/nm.2969

[87] L. Ding , T. L. Saunders , G. Enikolopov , S. J. Morrison , Nature 2012, 481, 457. 22281595 10.1038/nature10783 PMC3270376

[88] M. Skårn , P. Noordhuis , M.‐Y. Wang , M. Veuger , S. H. Kresse , E. V. Egeland , F. Micci , H. M. Namløs , A.‐M. Håkelien , S. M. Olafsrud , S. Lorenz , G. Haraldsen , G. Kvalheim , L. A. Meza‐Zepeda , O. Myklebost , Stem Cells Dev. 2014, 23, 2377. 24857590 10.1089/scd.2013.0599 PMC4172386

[89] S. Hauser , F. Jung , J. Pietzsch , Trends Biotechnol. 2017, 35, 265. 27789063 10.1016/j.tibtech.2016.09.007

[90] J. M. Butler , E. J. Gars , D. J. James , D. J. Nolan , J. M. Scandura , S. Rafii , Blood 2012, 120, 1344. 22709690 10.1182/blood-2011-12-398115 PMC3418723

[91] S. S. Kotha , B. J. Hayes , K. T. Phong , M. A. Redd , K. Bomsztyk , A. Ramakrishnan , B. Torok‐Storb , Y. Zheng , Stem Cell Res. Ther. 2018, 9, 77. 29566751 10.1186/s13287-018-0808-2 PMC5865379

[92] V. Barnhouse , N. Petrikas , C. Crosby , J. Zoldan , B. Harley , Ann. Biomed. Eng. 2021, 49, 780. 32939609 10.1007/s10439-020-02602-0 PMC7854499

[93] T. Bessy , T. Itkin , D. Passaro , Front. Cell Dev. Biol. 2021, 9, 645496. 33996805 10.3389/fcell.2021.645496 PMC8113773

[94] W. C. Aird , Circ. Res. 2007, 100, 158. 17272818 10.1161/01.RES.0000255691.76142.4a

[95] S. Rafii , J. M. Butler , B.‐S. Ding , Nature 2016, 529, 316. 26791722 10.1038/nature17040 PMC4878406

[96] D. Bouïs , G. A. Hospers , C. Meijer , G. Molema , N. H. Mulder , Angiogenesis 2001, 4, 91. 11806248 10.1023/a:1012259529167

[97] J.‐T. Chi , H. Y. Chang , G. Haraldsen , F. L. Jahnsen , O. G. Troyanskaya , D. S. Chang , Z. Wang , S. G. Rockson , M. van de Rijn , D. Botstein , P. O. Brown , Proc. Natl. Acad. Sci. U. S. A. 2003, 100, 10623. 12963823 10.1073/pnas.1434429100 PMC196854

[98] M. Urbanczyk , A. Zbinden , K. Schenke‐Layland , Adv. Drug Delivery Rev. 2022, 186, 114323. 10.1016/j.addr.2022.114323 35568103

[99] F. Arai , K. Hosokawa , H. Toyama , Y. Matsumoto , T. Suda , Ann. N. Y. Acad. Sci. 2012, 1266, 72. 22901259 10.1111/j.1749-6632.2012.06576.x

[100] J.‐P. Levesque , I. G. Winkler , Curr. Stem Cell Rep. 2016, 2, 356.

[101] K.‐N. Chua , C. Chai , P.‐C. Lee , Y.‐N. Tang , S. Ramakrishna , K. W. Leong , H.‐Q. Mao , Biomaterials 2006, 27, 6043. 16854459 10.1016/j.biomaterials.2006.06.017

[102] K.‐N. Chua , C. Chai , P.‐C. Lee , S. Ramakrishna , K. W. Leong , H.‐Q. Mao , Exp. Hematol. 2007, 35, 771. 17577926 10.1016/j.exphem.2007.02.002 PMC2376815

[103] D. Reichert , J. Friedrichs , S. Ritter , T. Käubler , C. Werner , M. Bornhäuser , D. Corbeil , Sci. Rep. 2015, 5, 15680. 26498381 10.1038/srep15680 PMC4620509

[104] A.‐V. Fonseca , D. Freund , M. Bornhäuser , D. Corbeil , J. Biol. Chem. 2010, 285, 31661. 20682776 10.1074/jbc.M110.145037 PMC2951238

[105] T. Bessy , A. Candelas , B. Souquet , K. Saadallah , A. Schaeffer , B. Vianay , D. Cuvelier , S. Gobaa , C. Nakid‐Cordero , J. Lion , J.‐C. Bories , N. Mooney , T. Jaffredo , J. Larghero , L. Blanchoin , L. Faivre , S. Brunet , M. Théry , J. Cell Biol. 2021, 220, 202005085. 10.1083/jcb.202005085 PMC8479938 34570198

[106] P. A. McKee , P. Mattock , R. L. Hill , Proc. Natl Acad. Sci. USA 1970, 66, 738. 5269236 10.1073/pnas.66.3.738 PMC283112

[107] H. P. Erickson , N. Carrell , J. McDonagh , J. Cell Biol. 1981, 91, 673. 7328116 10.1083/jcb.91.3.673 PMC2112785

[108] S. Stamboroski , A. Joshi , P.‐L. M. Noeske , S. Köppen , D. Brüggemann , Macromol. Biosci. 2021, 21, 2000412. 10.1002/mabi.202000412 33687802

[109] C. Ota , Y. Fukuda , S.‐I. Tanaka , K. Takano , Langmuir 2020, 36, 14243. 33197316 10.1021/acs.langmuir.0c02367

[110] J. Sánchez‐Cortés , M. Mrksich , Chem. Biol. 2009, 16, 990. 19778727 10.1016/j.chembiol.2009.08.012 PMC2788794

[111] S. D. Redick , D. L. Settles , G. Briscoe , H. P. Erickson , J. Cell Biol. 2000, 149, 521. 10769040 10.1083/jcb.149.2.521 PMC2175162

[112] A. J. Zollinger , M. L. Smith , Matrix Biol. 2017, 60‐61, 27. 10.1016/j.matbio.2016.07.011 27496349

[113] C. Fuss , J. C. Palmaz , E. A. Sprague , J. Vasc. Interventional Radiol. 2001, 12, 677. 10.1016/s1051-0443(07)61437-7 11389218

[114] R. Smith , M. Rooney , S. Lord , M. W. Mosesson , T. Gartner , Thromb. Haemostatis 2000, 84, 819. 11127863

[115] K. Anselme , M. Bigerelle , Int. Mater. Rev. 2011, 56, 243.

[116] Y. Hou , W. Xie , L. Yu , L. C. Camacho , C. Nie , M. Zhang , R. Haag , Q. Wei , Small 2020, 16, 1905422. 10.1002/smll.201905422 32064782

[117] A. M. Ross , Z. Jiang , M. Bastmeyer , J. Lahann , Small 2012, 8, 336. 22162324 10.1002/smll.201100934

[118] M. Schindler , I. Ahmed , J. Kamal , A. Nur‐E‐Kamal , T. H. Grafe , H. Y. Chung , S. Meiners , Biomaterials 2005, 26, 5624. 15878367 10.1016/j.biomaterials.2005.02.014

[119] Y.‐R. V. Shih , C.‐N. Chen , S.‐W. Tsai , Y. J. Wang , O. K. Lee , Stem Cells 2006, 24, 2391. 17071856 10.1634/stemcells.2006-0253

[120] Y. Zhang , X. Wang , Y. Zhang , Y. Liu , D. Wang , X. Yu , H. Wang , Z. Bai , Y. Jiang , X. Li , W. Zheng , Q. Li , ACS Biomater. Sci. Eng. 2021, 7, 4959. 34543012 10.1021/acsbiomaterials.1c00951

[121] R. M. Lemoli , A. D'Addio , Haematologica 2008, 93, 321. 18310535 10.3324/haematol.12616

[122] A. Burberry , M. Y. Zeng , L. Ding , I. Wicks , N. Inohara , S. J. Morrison , G. Núñez , Cell Host Microbe 2014, 15, 779. 24882704 10.1016/j.chom.2014.05.004 PMC4085166

[123] A. Herman , M. Romine , D. Monlish , L. G. Schuettpelz , Blood 2014, 124, 4334.

[124] M. Z. Ratajczak , M. Adamiak , M. Plonka , A. Abdel‐Latif , J. Ratajczak , Leukemia 2018, 32, 1116. 29556022 10.1038/s41375-018-0087-z PMC5940655

[125] J. L. Granick , S. I. Simon , D. L. Borjesson , Bone Marrow Res. 2012, 2012, 165107. 22762001 10.1155/2012/165107 PMC3385697

[126] T. Hirano , Int. Immunol. 2021, 33, 127. 33337480 10.1093/intimm/dxaa078 PMC7799025

[127] S. Singh , D. Anshita , V. Ravichandiran , Int. Immunopharmacol. 2021, 101, 107598. 34233864 10.1016/j.intimp.2021.107598 PMC8135227

[128] K. Matsushima , D. Yang , J. J. Oppenheim , Cytokine 2022, 153, 155828. 35247648 10.1016/j.cyto.2022.155828

[129] S. Aggarwal , M. F. Pittenger , Blood 2005, 105, 1815. 15494428 10.1182/blood-2004-04-1559

[130] I. B. Copland , M. A. Garcia , E. K. Waller , J. D. Roback , J. Galipeau , Biomaterials 2013, 34, 7840. 23891515 10.1016/j.biomaterials.2013.06.050

[131] D. M. Vasconcelos , R. M. Gonçalves , C. R. Almeida , I. O. Pereira , M. I. Oliveira , N. Neves , A. M. Silva , A. C. Ribeiro , C. Cunha , A. R. Almeida , C. C. Ribeiro , A. M. Gil , E. Seebach , K. L. Kynast , W. Richter , M. Lamghari , S. G. Santos , M. A. Barbosa , Biomaterials 2016, 111, 163. 27728815 10.1016/j.biomaterials.2016.10.004

[132] L. Shi , L. Shi , X. Wang , J. He , Cell Transplant. 2018, 27, 1185. 30001635 10.1177/0963689718756070 PMC6434466

[133] W. Zheng , R. Li , H. Pan , D. He , R. Xu , T. B. Guo , Y. Guo , J. Z. Zhang , Arthritis Rheum. 2009, 60, 1957. 19565503 10.1002/art.24625

[134] K.‐W. Kang , S.‐J. Lee , J. H. Kim , B.‐H. Lee , S. J. Kim , Y. Park , B. S. Kim , BMC Cancer 2020, 20, 619. 32615949 10.1186/s12885-020-07102-x PMC7330970

[135] S. G. Almalki , D. K. Agrawal , Stem Cell Res. Ther. 2016, 7, 129. 27612636 10.1186/s13287-016-0393-1 PMC5016871

[136] X. Wang , R. A. Khalil , Adv. Pharmacol. 2018, 81, 241. 29310800 10.1016/bs.apha.2017.08.002 PMC5765875

[137] O. Hiller , A. Lichte , A. Oberpichler , A. Kocourek , H. Tschesche , J. Biol. Chem. 2000, 275, 33008. 10930399 10.1074/jbc.M001836200

[138] J. Orbe , J. Barrenetxe , J. A. Rodriguez , D. Vivien , C. Orset , W. C. Parks , T. P. Birkland , R. Serrano , A. Purroy , S. Martinez de Lizarrondo , E. Angles‐Cano , J. A. Páramo , Circulation 2011, 124, 2909. 22104553 10.1161/CIRCULATIONAHA.111.047100

[139] L. Kumar , J. Planas‐Iglesias , C. Harms , S. Kamboj , D. Wright , J. Klein‐Seetharaman , S. K. Sarkar , Sci. Rep. 2020, 10, 20615. 33244162 10.1038/s41598-020-77699-3 PMC7692495

[140] V. Arpino , M. Brock , S. E. Gill , Matrix Biol. 2015, 44–46, 247. 10.1016/j.matbio.2015.03.005 25805621

[141] L. Costanzo , B. Soto , R. Meier , P. Geraghty , Pulm. Med. 2022, 2022, 3632764. 36624735 10.1155/2022/3632764 PMC9825218

[142] T. P. Lozito , R. S. Tuan , J. Cell. Physiol. 2011, 226, 385. 20665704 10.1002/jcp.22344

[143] J. Liu , J. Gao , Z. Liang , C. Gao , Q. Niu , F. Wu , L. Zhang , Stem Cell Res. Ther. 2022, 13, 429. 35987711 10.1186/s13287-022-02985-y PMC9391632

[144] Y. Han , J. Yang , J. Fang , Y. Zhou , E. Candi , J. Wang , D. Hua , C. Shao , Y. Shi , Signal Transduction Targeted Ther. 2022, 7, 92. 10.1038/s41392-022-00932-0 PMC8935608 35314676

[145] C. M. Trigo , J. S. Rodrigues , S. P. Camões , S. Solá , J. P. Miranda , J. Adv. Res. 2025, 70, 103. 38729561 10.1016/j.jare.2024.05.004 PMC11976416

[146] W. Wagner , F. Wein , C. Roderburg , R. Saffrich , A. Faber , U. Krause , M. Schubert , V. Benes , V. Eckstein , H. Maul , A. D. Ho , Exp. Hematol. 2007, 35, 314. 17258080 10.1016/j.exphem.2006.10.003

[147] E. V. Sotnezova , E. R. Andreeva , A. I. Grigoriev , L. B. Buravkova , Acta Naturae 2016, 8, 6. PMC5081707 27795840

[148] D. Jing , M. Wobus , D. M. Poitz , M. Bornhäuser , G. Ehninger , R. Ordemann , Haematologica 2012, 97, 331. 22058205 10.3324/haematol.2011.050815 PMC3291585

[149] D. Jing , A.‐V. Fonseca , N. Alakel , F. A. Fierro , K. Muller , M. Bornhauser , G. Ehninger , D. Corbeil , R. Ordemann , Haematologica 2010, 95, 542. 20145267 10.3324/haematol.2009.010736 PMC2857183

[150] A. O. Sahin , M. Buitenhuis , Cell Adhes. Migr. 2012, 6, 39. 10.4161/cam.18975 PMC3364136 22647939

[151] T. Rademakers , M. Goedhart , M. Hoogenboezem , A. García Ponce , J. van Rijssel , M. Samus , M. Schnoor , S. Butz , S. Huveneers , D. Vestweber , M. A. Nolte , C. Voermans , J. D. van Buul , Haematologica 2020, 105, 2746. 33256374 10.3324/haematol.2018.196329 PMC7716366

[152] N. He , L. Zhang , J. Cui , Z. Li , Bone Marrow Res. 2014, 2014, 128436. 24822129 10.1155/2014/128436 PMC4009113

[153] G. Sauvageau , N. N. Iscove , R. K. Humphries , Oncogene 2004, 23, 7223. 15378082 10.1038/sj.onc.1207942

[154] A. Branco , S. Bucar , J. Moura‐Sampaio , C. Lilaia , J. M. S. Cabral , A. Fernandes‐Platzgummer , C. Da Lobato Silva , Front. Bioeng. Biotechnol. 2020, 8, 573282. 33330414 10.3389/fbioe.2020.573282 PMC7729524

[155] E. Zimran , L. Papa , R. Hoffman , Blood Rev. 2021, 50, 100853. 34112560 10.1016/j.blre.2021.100853

[156] S. Bastani , F. J. T. Staal , K. Canté‐Barrett , Stem Cell Investig. 2023, 10, 15. 10.21037/sci-2023-016 PMC10345135 37457748

[157] M. R. Koller , I. Manchel , D. A. Brott , B. Palsson , Exp. Hematol. 1996, 24, 1484. 8950231

[158] M. E. Belderbos , S. Jacobs , T. K. Koster , A. Ausema , E. Weersing , E. Zwart , G. de Haan , L. V. Bystrykh , Biol. Blood Marrow Transplant. 2020, 26, 16. 31494231 10.1016/j.bbmt.2019.08.026

[159] L. Wang , X. Guan , H. Wang , B. Shen , Y. Zhang , Z. Ren , Y. Ma , X. Ding , Y. Jiang , Stem Cell Res. Ther. 2017, 8, 169. 28720126 10.1186/s13287-017-0625-z PMC5516306

[160] C. Winstead , L. Lott , K. Milligan , L. Hyppolite , Med. Res. Arch. 2020, 8.

[161] S. K. Nilsson , H. M. Johnston , G. A. Whitty , B. Williams , R. J. Webb , D. T. Denhardt , I. Bertoncello , L. J. Bendall , P. J. Simmons , D. N. Haylock , Blood 2005, 106, 1232. 15845900 10.1182/blood-2004-11-4422

[162] S. Stier , Y. Ko , R. Forkert , C. Lutz , T. Neuhaus , E. Grünewald , T. Cheng , D. Dombkowski , L. M. Calvi , S. R. Rittling , D. T. Scadden , J. Exp. Med. 2005, 201, 1781. 15928197 10.1084/jem.20041992 PMC2213260

[163] L. Rossi , D. Forte , G. Migliardi , V. Salvestrini , M. Buzzi , M. R. Ricciardi , R. Licchetta , A. Tafuri , S. Bicciato , M. Cavo , L. Catani , R. M. Lemoli , A. Curti , Exp. Hematol. 2015, 43, 974.e1. 26213230 10.1016/j.exphem.2015.07.003

[164] S. Aqmasheh , K. Shamsasanjan , P. Akbarzadehlaleh , D. Pashoutan Sarvar , H. Timari , Adv. Pharm. Bull. 2017, 7, 165. 28761818 10.15171/apb.2017.021 PMC5527230

[165] J. Schindelin , I. Arganda‐Carreras , E. Frise , V. Kaynig , M. Longair , T. Pietzsch , S. Preibisch , C. Rueden , S. Saalfeld , B. Schmid , J.‐Y. Tinevez , D. J. White , V. Hartenstein , K. Eliceiri , P. Tomancak , A. Cardona , Nat. Methods 2012, 9, 676. 22743772 10.1038/nmeth.2019 PMC3855844

[166] S. L. Meermeyer , PhD Thesis , Leibniz Universität Hannover, 2024.

