# Supplementary material for: Biomimetic Fibrinogen Nanofiber Scaffolds for Vascular Hematopoietic Stem Cell Niche Engineering
Source: Adv Healthc Mater. 2025 Oct 28;15(5):e03449. doi: 10.1002/adhm.202503449 (PMC12864591; doi:10.1002/adhm.202503449)
Supplement: Supplementary file 1 — Supporting Information: Supporting figures, tables, methods, description of supporting videos. [file ADHM-15-0-s001.pdf]

ADVANCED HEALTHCARE MATERIALS

Supporting Information

for *Adv. Healthcare Mater.*, DOI 10.1002/adhm.202503449

Biomimetic Fibrinogen Nanofiber Scaffolds for Vascular Hematopoietic Stem Cell Niche Engineering

*Sophia Lena Meermeyer, Arundhati Joshi, Constantin von Kaisenberg, Dorothea Brüggemann and Cornelia Lee-Thedieck**

Supporting Information

Biomimetic Fibrinogen Nanofiber Scaffolds for Vascular Hematopoietic Stem Cell Niche Engineering

*Sophia Lena Meermeyer, Arundhati Joshi, Constantin von Kaisenberg, Dorothea Brüggemann, Cornelia Lee-Thedieck**

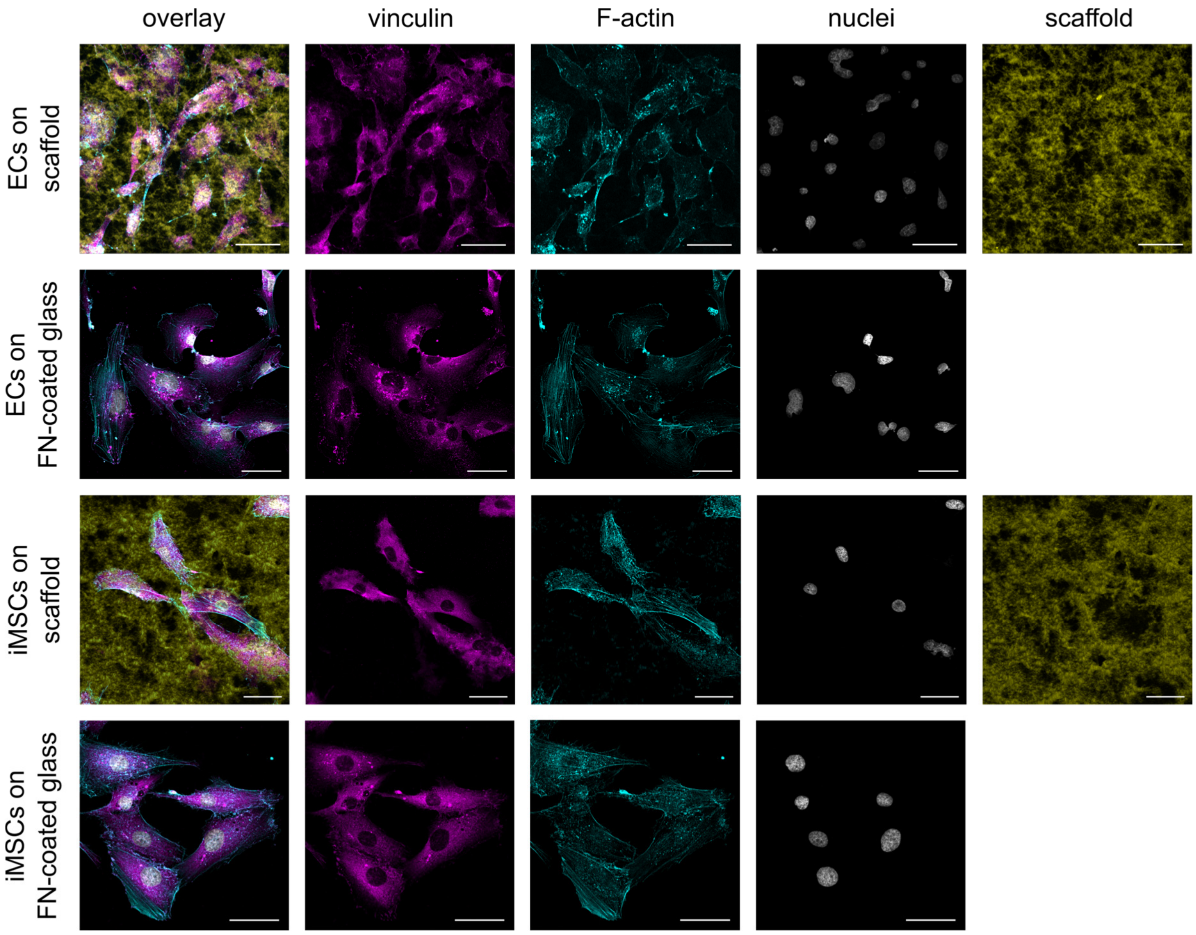

Figure S1. Adhesion of HMEC-1 (ECs) and iMSC#3 cells (iMSCs) to immobilized fibrinogen scaffolds in comparison to adhesion to FN-coated glass coverslips visualized by vinculin staining. The cells were cultured for a period of 4 days, subsequently IF stained and analyzed via microscopy. Z-stacks of the culture conditions (described on the left) were captured (4 per condition) and maximum projection images were created. Images of the different channels are depicted in columns. In the first column, overlay images of all channels are given for every culture condition, followed by single channel images of the vinculin staining (pink), F-actin staining (turquoise), nucleus staining (white) and scaffold autofluorescence (yellow). The images were taken with a cLSM in the 20x objective using the Airyscan detector in the multiplex mode (CO-8Y for cultures on scaffolds, SR-4Y for cultures on FN-coated glass). The scale bars represent 50 µm.

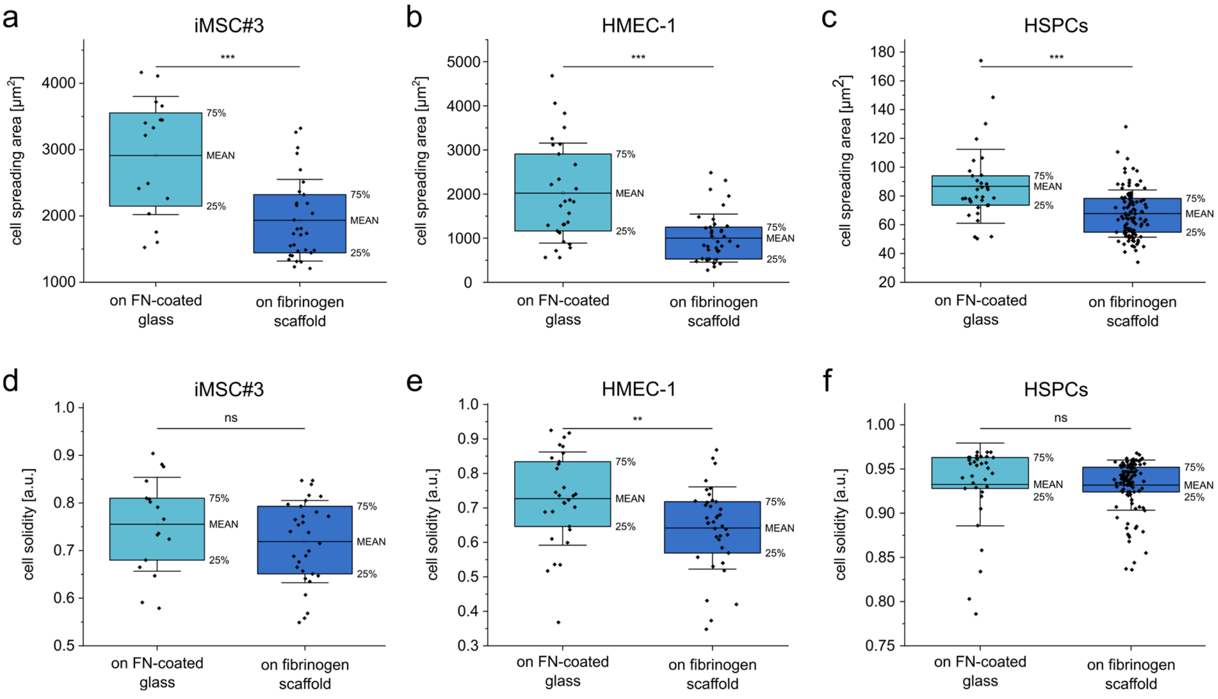

Figure S2. Cells possessed a higher spreading area and lower solidity on immobilized fibrinogen scaffolds compared to FN-coated glass. The analysis was performed on microscopy images of single cultures of iMSC#3, HMEC-1 and HSPCs on both materials. (a) - (c) show the cell spreading area and (d) - (f) the cell solidity. The cells were cultured for a period of 4 days, subsequently IF stained and submitted to microscopy. Cell spreading area and solidity was determined from the F-actin staining using Fiji. For every cell type, 4 images on each material were analyzed. Each data point represents the data of one cell. The mean is indicated by a line, the whiskers represent the SD and the 25- and 75-percentile are given by the edges of the box. For statistical analysis, a t-test was performed (*: $p \leq 0.05$, **: $p \leq 0.01$, ***: $p \leq 0.001$, ns: not significant).

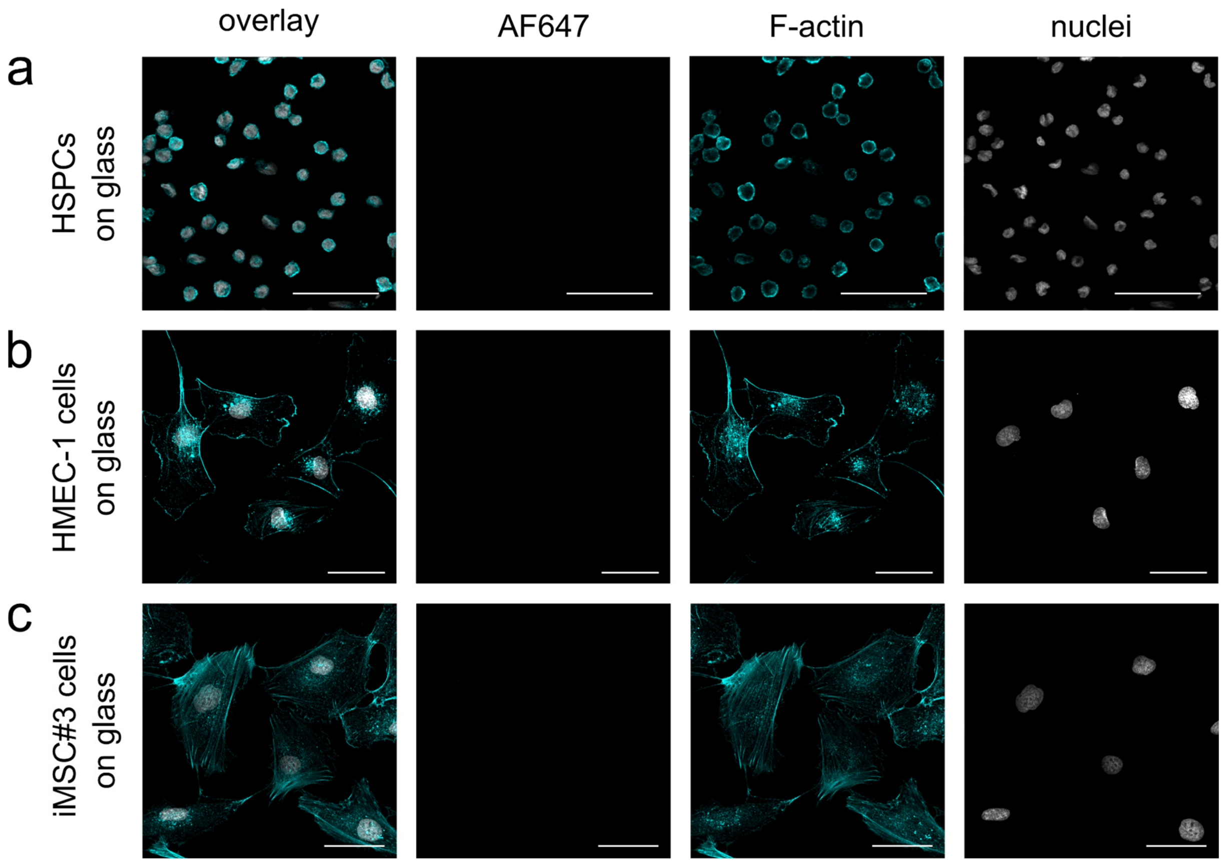

Figure S3. Representative images of negative IF staining control omitting the primary AB for the vinculin staining of cells grown on immobilized fibrinogen scaffolds. HSPCs, HMEC-1 and iMSC#3 cells on fibronectin-coated glass coverslips were stained after a culture period of 4 days. (a-c) show the staining of the different cell types and single channel images are depicted in columns. In the first column, an overlay image of all channels is given for every cell type. The second column shows the channels of the staining with the secondary AB (AF647, mouse, pink). In the next columns, the F-actin (turquoise) and nuclei (white) staining is shown. The images were taken with a cLSM in the 20x objective using the Airyscan detector in the multiplex mode (SR-4Y). The scale bars represent 50 µm.

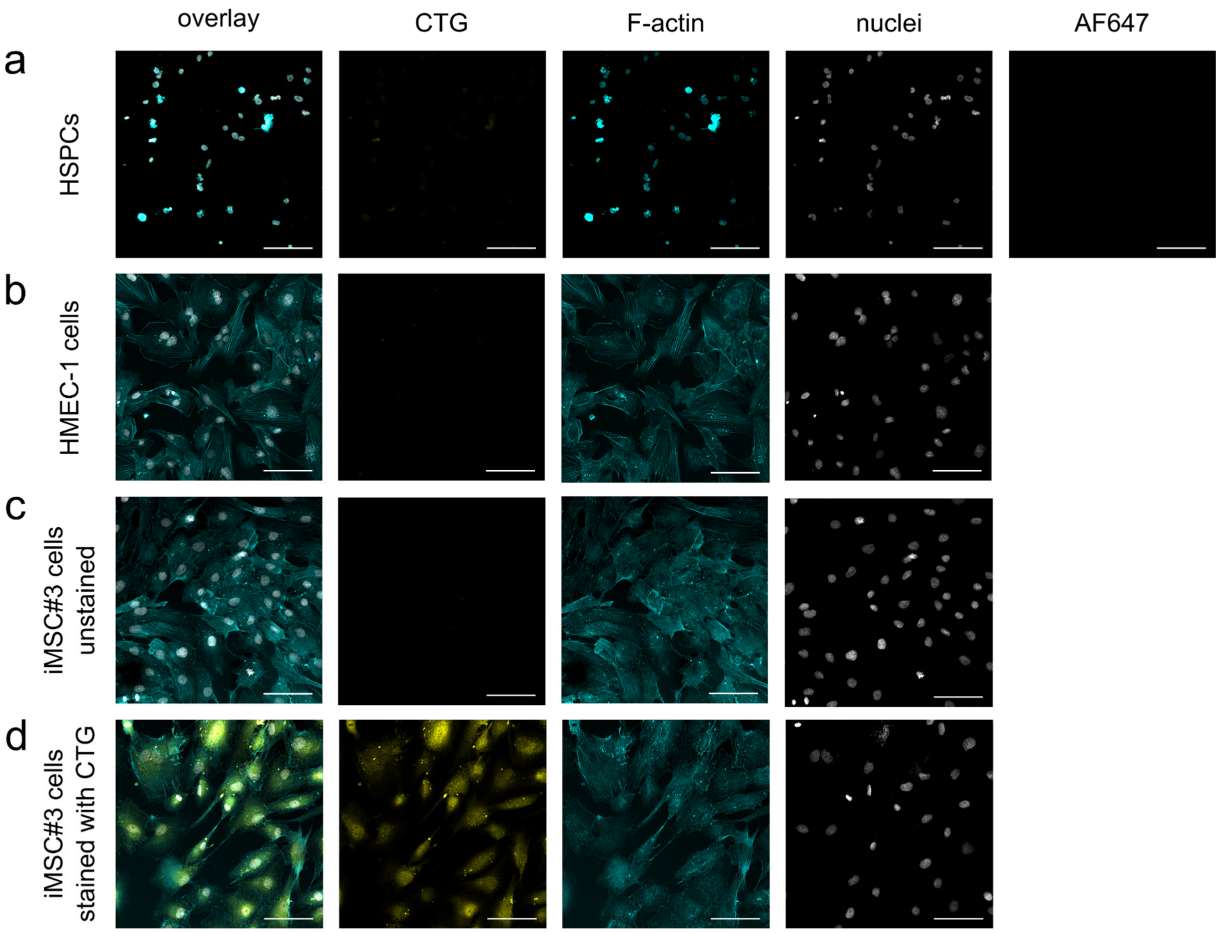

Figure S4. Representative images of negative IF staining controls containing only secondary ABs for experiments on free-standing fibrinogen scaffold. HSPCs, HMEC-1 and iMSC#3 cells on fibronectin-coated glass coverslips were stained after a culture period of 5 days. (A-D) show the staining of the different cell types and single channel images are depicted in columns. In the first column, an overlay image of all channels is given for every cell type. The second column shows the CTG staining of the iMSC#3 cells (yellow). The next columns show the F-actin (turquoise) and nuclei (white) staining. In the last column, the channel of the staining with the secondary AB (AF647, rat, pink) is shown. The images were taken with a cLSM in the 20x objective using the Airyscan detector in the multiplex mode (SR-4Y). The scale bars represent 100 µm. CTG: CellTracker™ Green.

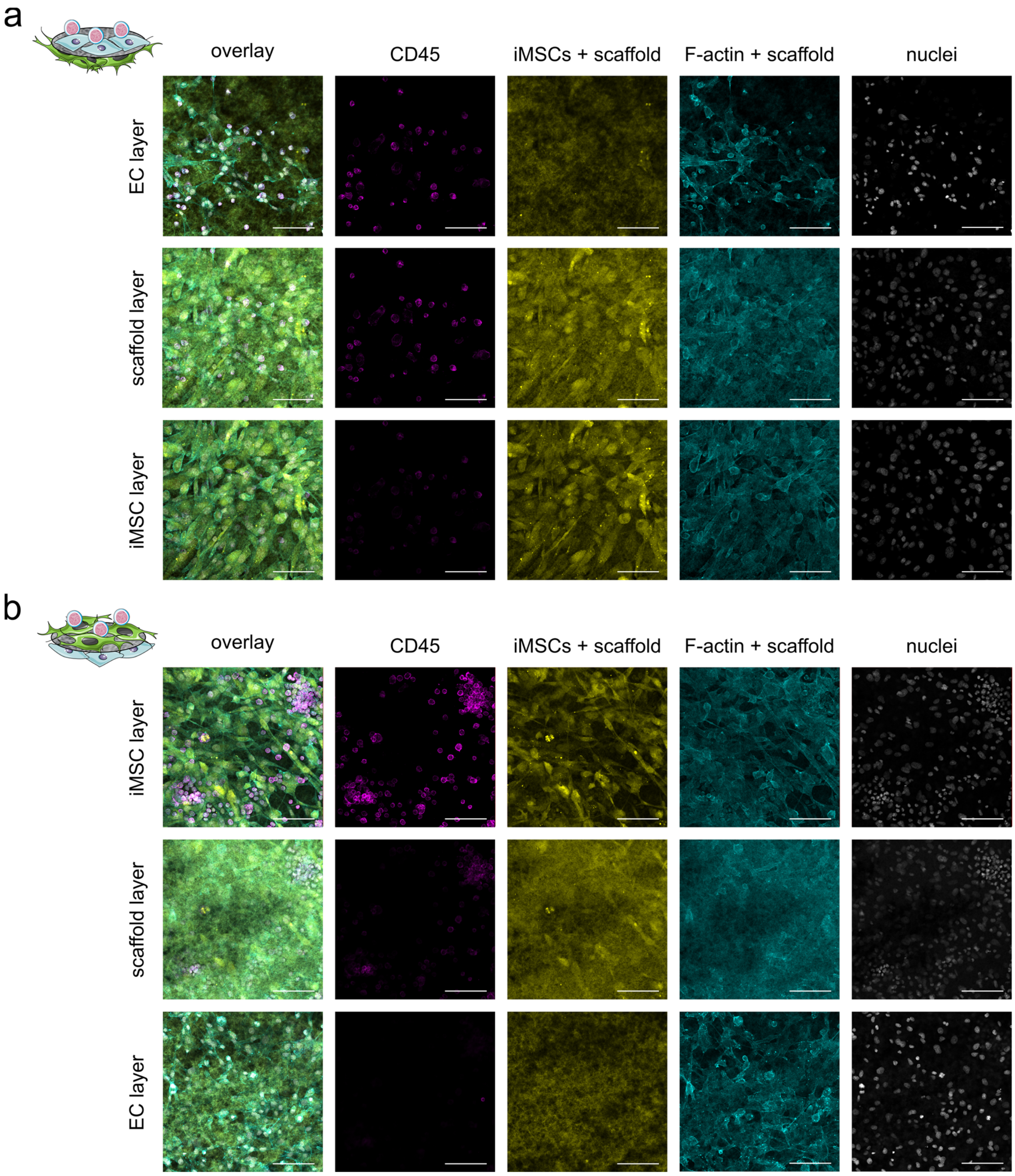

Figure S5. Spatial localization, interaction and morphology of HSPCs, HMEC-1 and iMSC#3 cells in triple co-cultures on free-standing fibrinogen scaffolds. The difference between the triple co-cultures is the HSPC seeding side. In a) HSPCs were seeded on top of HMEC-1 and in b) on top of iMSC#3 cells. The cells were cultured for a period of 5 days, subsequently IF stained and analyzed via microscopy. As the triple co-cultures consist of different layers, z-stacks were captured. For both triple co-culture conditions, three rows of representative images are displayed showing EC-, scaffold- and iMSC#3-layer. Images of the different channels are depicted in columns. In the first column, overlay images of all channels are given for every culture condition, followed by images of the CD45 staining (pink), iMSC#3 staining and scaffold autofluorescence (yellow), F-actin staining and scaffold autofluorescence (turquoise) and nucleus staining (white). Four independent experiments, each containing two technical replicates of every condition, were performed. The images (4 per condition in each experiment) were taken with a cLSM in the 10x objective using the Airyscan detector in the multiplex mode (SR-4Y). The scale bar represents 100 µm.

Spatial distribution of cells on scaffolds - videos

To obtain an impression of the spatial distribution of the cells on the scaffolds in all experimental conditions, 3D reconstruction videos as well as videos of z-stacks containing all z-layers were created (supplementary videos 1-5). Each video consists of a 3D reconstruction (maximum intensity projection mode + transparency mode) and a z-stack based on the same microscopy images. The transparency mode provides additional insights as it facilitates the visualization of the cell locations on both sides of the scaffold. For the video preparation, images of the CD45 staining (pink), iMSC#3 cell tracker staining (yellow), F-actin staining (turquoise) and nucleus staining (white) were taken. Additionally, the scaffold autofluorescence is visible in yellow and turquoise. For clarity, the nucleus staining was omitted from the 3D reconstruction videos. Four independent experiments, each containing two technical replicates of every condition, were performed. The z-stacks (4 per condition in each experiment) were taken with a cLSM in the 10x objective using the Airyscan detector in the multiplex mode (SR 4Y).

Supplementary video 1: Scaffold, ECs + HSPCs
Supplementary video 2: iMSCs, scaffold, ECs + HSPCs
Supplementary video 3: ECs, scaffold, iMSCs + HSPCs
Supplementary video 4: Scaffold, iMSCs + HSPCs
Supplementary video 5: Scaffold, HSPCs

Table S1: HSPC numbers after a culture period of 5 days on free-standing fibrinogen scaffolds. Numbers represent fold changes in regard to the initial seeding number of 10 000 HSPCs per condition. Fold changes of four experiments were averaged.

Experimental condition	HSPC fold change
Scaffold, ECs + HSPCs	41.06
iMSCs, scaffold, ECs + HSPCs	49.75
ECs, scaffold, iMSCs + HSPCs	44.13
Scaffold, iMSCs + HSPCs	29.50
Scaffold, HSPCs	28.69
HPSCs on TCPS	35.00

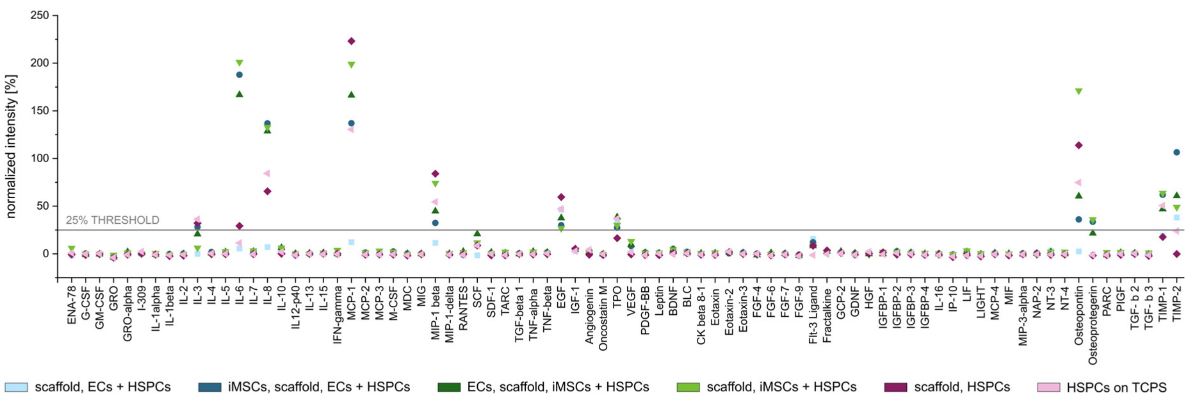

Figure S6. Detected cytokines in the supernatant of experimental conditions. A membrane-based cytokine array was used to analyze medium supernatants after the culture of HSPCs, HMEC-1 and iMSC#3 cells for a period of 5 days on free-standing fibrinogen scaffolds. Supernatants of four independent experiments were pooled for the array. The x-axis shows the tested cytokines and on the y-axis the intensity, which was normalized to an internal positive control is given. A 25% threshold (grey line) was set to discriminate signals from background noise.

Detachment and clamping of fibrinogen scaffolds

Handling of the detached scaffolds needed to be done with special care, as they could easily disrupt. For transferring and touching the detached scaffolds, polybutene terephthalate (PBTP) forceps with straight, flat wide tips (K35A, rubis, Stabio, Switzerland) were used. To handle the clamped scaffolds, stainless steel forceps with curved, pointed tips were optimally suited as they allow lowering of the rings in a tilted way towards the prepared wells. This helped to avoid disruptions of the scaffolds as well as air bubbles underneath the scaffold.

The detachment and clamping process is described and shown in Figure S7 as well as in supplementary video 6. All materials were sterilized before use by overnight immersion in 70% EtOH. In the first step, a sterilized scaffold (UV, 30 min) was transferred into a 35 mm petri dish with sterile ddH_2O (Figure S7, 1). Using a 1000 µL pipette, the scaffold was carefully detached by flushing it from the glass coverslip (Figure S7, 2). The free-standing scaffold was clamped using two PVC rings (outer diameter: 30 mm, inner diameter: 10 mm, thickness: 2 mm). For assembling the rings, plastics screws, stainless steel nuts and a 3D printed hexagon spanner were used. In preparation for scaffold clamping, the lower PVC ring containing the screws was placed in a 90 mm petri dish and covered with ddH_2O. Then, the detached scaffold was carefully transferred to this second petri dish using the PBTP forceps. By slightly touching the scaffold with the forceps, it was centered above the PVC ring (Figure S7, 3). To settle the scaffold onto the ring, the water level was decreased using a Pasteur

pipette connected to a vacuum pump (Figure S7, 3). Subsequently, the second ring was placed on top of the settled scaffold (Figure S7, 4) and three nuts were placed onto the screws using forceps (Figure S7, 5). Both rings were screwed together with the 3D printed hexagon spanner (Figure S7, 6-7). Finally, the clamped scaffold was transferred to a 6 well plate with sterile ddH_2O (curved forceps, Figure S7, 9) and stored in the incubator until the next day.

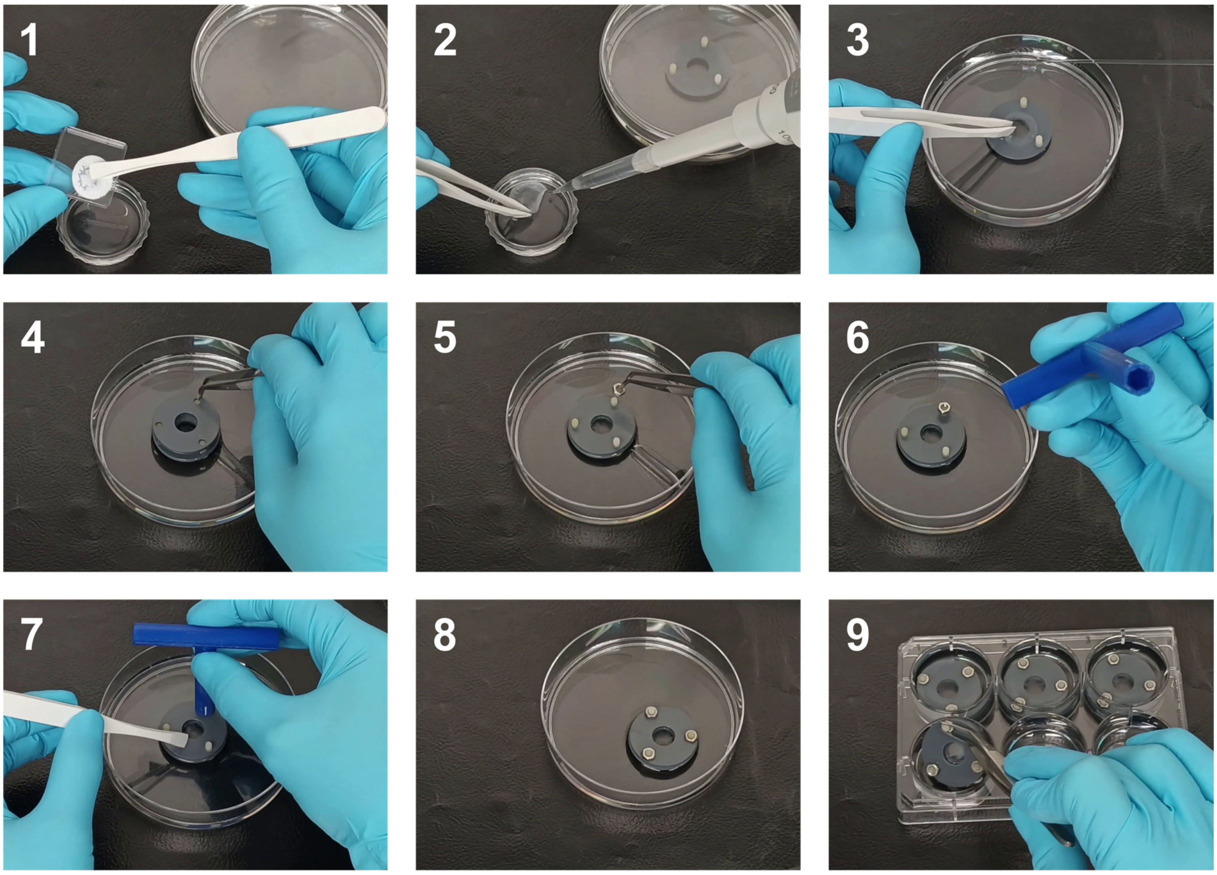

Figure S7. Detachment and clamping of detached fibrinogen scaffolds. (1) Transfer of the scaffold into a petri dish with sterile ddH_2O. (2) Detachment of the scaffold by flushing it from the glass coverslip using a pipette. (3) By slightly touching the scaffold with the forceps, it is centered above the lower PVC ring. To settle the scaffold onto the ring, the water level is decreased using a Pasteur pipette, which is connected to a vacuum pump. (4) Placement of the second PVC ring on top of the settled scaffold. (5) A nut is placed onto the screw using forceps. (6), (7) A 3D printed hexagon spanner is used to fasten the nuts. (8) The completely clamped scaffold. (9) The clamped scaffold is transferred into a 6 well plate with ddH2O using forceps. PVC: polyvinyl chloride. A video of the detachment and clamping process is shown in supplementary video 6.

Seeding of cells on clamped fibrinogen scaffolds

The seeding procedure is shown in Figure S8 and in the supplementary video 7. For cell seeding, small silicone rings (two-component duplicating silicone (Replisil 22 N A+B, Siltecs, Ulm, Germany), outer diameter: 14 mm, inner diameter: 10 mm, height: 6-8 mm) were placed onto the PVC rings. To prepare for the seeding of the HMEC-1 cells, three wells of a 6 well plate were filled with HMEC-1 medium. Then, three clamped scaffolds were transferred into the prepared wells using forceps (Figure S8, 1) and silicone rings were placed on top of the PVC rings for cell seeding (Figure S8, 2). The HMEC-1 cells were detached from the cell culture flask and counted. 150 000 cells were seeded into each silicone ring in 100 µL of medium (Figure S8, 3). Subsequently, 0.5 - 1 mL of medium was added to every scaffold to ensure sufficient coverage with medium and the seeded scaffolds were incubated for 3 h at 37 °C and 5% CO_2. The iMSC#3 cells were stained with CTG prior to seeding onto the scaffolds to enable discrimination between cell types. The dye was reconstituted according to the manufacturers' instructions. From the obtained 10 mM stock solution, a staining solution with a concentration of 10 µM was prepared using alpha minimum essential medium (αMEM) without FBS. To facilitate the intake of the dye, the iMSC#3 cells only had a confluency of 50 to 60%. For the CTG staining, the cell culture medium was removed and the prepared staining solution was added to the culture flask. Subsequently, the cells were incubated for 30 min at 37 °C and 5% CO_2. After the staining, the iMSC#3 cells were always handled in the dark to avoid photobleaching. The staining solution was replaced by normal culture medium and the flask was incubated at 37 °C and 5% CO_2 until seeding of the iMSC#3 cells. 3 h after seeding the HMEC-1 cells, the silicone rings were removed (Figure S8, 4) and the clamped scaffolds were transferred into an empty 6 well plate (Figure S8, 5). The HMEC-1 medium was discarded and all wells were filled according to the scheme in Fig. 2.13 (Figure S8, 6). The clamped scaffolds were turned and placed into the prepared well plate using forceps (Figure S8, 7-9). Consequently, the cell-seeded side of the scaffolds was facing the well bottom. Additionally, two unseeded clamped scaffolds were added to the well plate (Figure S8, 10). Then, silicone rings were placed on top of the PVC rings for cell seeding (Figure S8, 11). The CTG stained iMSC#3 cells were detached from the culture flask and counted. Subsequently, cell suspensions in the respective culture media were prepared and 10 000 cells in 100 µL were seeded to each silicone ring (Figure S8, 12). Afterwards, 0.5 - 1 mL of medium was added to every scaffold to ensure sufficient coverage with medium and the seeded scaffolds were incubated for 3 h at 37 °C and 5% CO_2. In a last seeding step, HSPCs were seeded onto the scaffolds. Beforehand, the percentage of $CD34^+$ cells was determined (flow cytometry). In

preparation for the HSPC seeding, five wells of a 6 well plate were filled with triple co-culture medium (TCM). Then, a silicone ring was placed into the 6th well (Figure S8, 13). 3 h after seeding of the iMSC#3 cells, the silicone rings on top of the clamped scaffolds were removed (Figure S8, 14). Subsequently, the rings with the scaffolds were turned (Figure S8, 15) and transferred into the prepared well plate (Figure S8, 16) in a way that the HSPC seeding side was facing upwards (according to the different culture conditions, Figure 3, b). Then, new silicone rings were placed onto the PVC rings (Figure S8, 17) and 5 000 cells in 100 μL were seeded into each ring (Figure S8, 18). Subsequently, the seeded scaffolds were incubated at 37 °C and 5% CO_2 for five days.

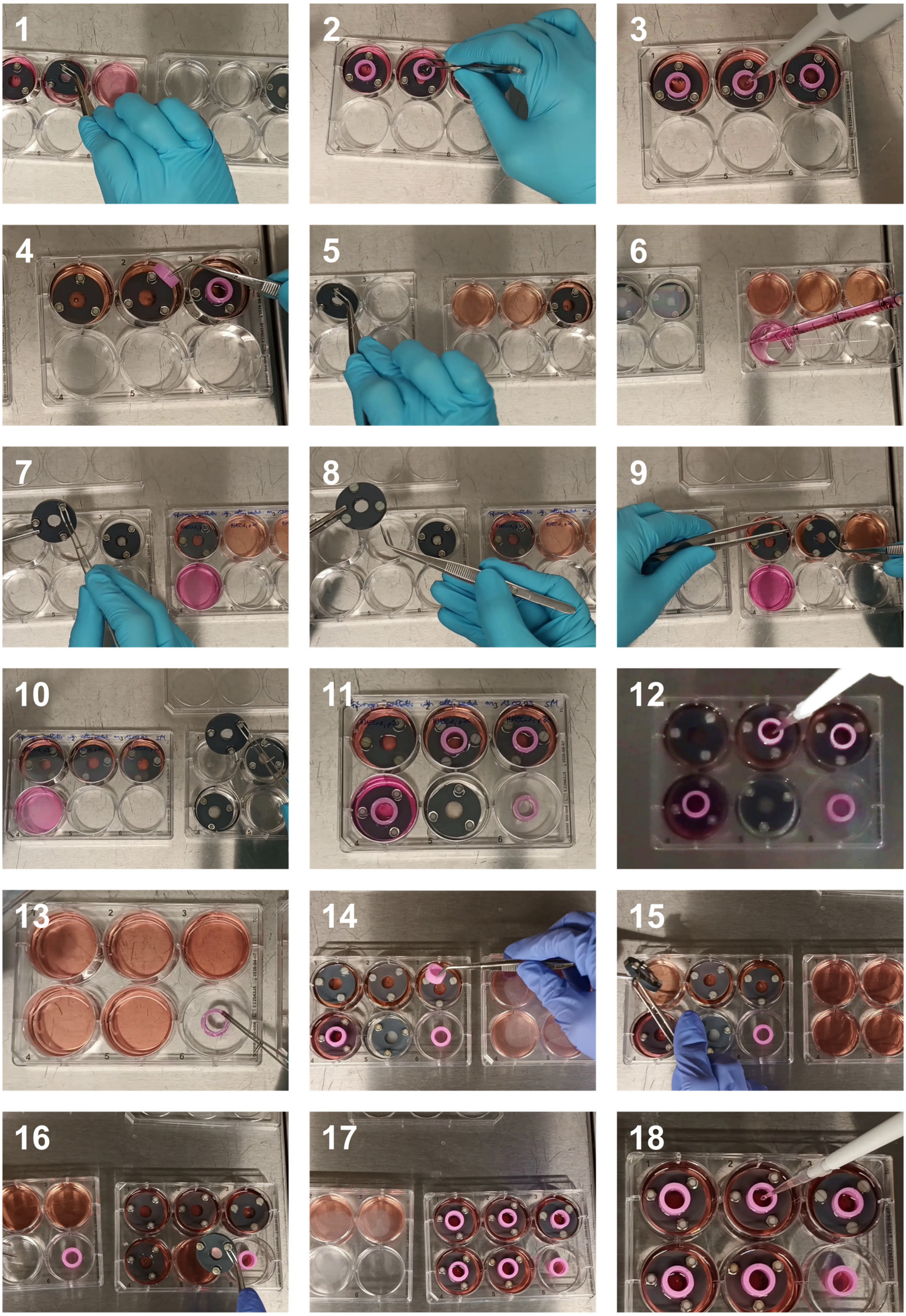

Figure S8. Cell seeding procedure on fibrinogen scaffolds. (1) Transfer of clamped scaffolds into the wells of a prepared medium-filled 6 well plate. (2) The silicone rings are placed on top

of the PVC rings for cell seeding. (3) Seeding of 15 000 HMEC-1 cells per silicone ring. (4) Removal of the silicone rings 3 h after seeding the HMEC 1 cells. (5) The clamped scaffolds are transferred into an empty 6 well plate. (6) Preparation of another 6 well plate with cell culture media for seeding of iMSC#3 cells. (7-9) Turning and transfer of the clamped scaffolds into the prepared well plate (cell-seeded side facing well bottom). (10) Two not seeded, clamped scaffolds are added to the well plate. (11) Placement of the silicone seeding rings on top of the PVC rings. (12) Seeding of 10 000 iMSC#3 cells per silicone ring. (13) Preparation of a 6 well plate for seeding of the HSPCs. (14) Removal of the silicone rings on top of the clamped scaffolds 3 h after seeding of the iMSC#3 cells. (15, 16) The rings with the scaffolds are turned and transferred into the prepared well plate (HSPC seeding side facing upwards). (17) Placement of new silicone rings onto the PVC rings for cell seeding. (18) Seeding of 5 000 HSPCs per silicone ring. PVC: polyvinyl chloride; HMEC-1: human microvascular endothelial cell line 1; iMSC#3: immortalized human bone marrow mesenchymal cell line; HSPCs: hematopoietic stem and progenitor cells. A video of the detachment and clamping process is shown in supplementary video 7.

Unclamping and mounting of fibrinogen scaffolds

The detailed seeding procedure is shown in Figure S9 as well as in supplementary video 8. In a first step, the clamped scaffold and a glass slide were transferred into a 90 mm petri dish with ddH$_2$O (Figure S9, 1). Then, the nuts were removed using a 3D printed hexagon spanner (Figure S9, 2-3) and the polyvinyl chloride (PVC) rings were carefully separated using forceps (Figure S9, 4, 5). After identifying to which of the PVC rings the scaffold was sticking, this ring needed to stay inside the water to avoid scaffold disruption. Then, the scaffold was carefully released from the ring using forceps (Figure S9, 6). Subsequently, the floating scaffold was placed above the glass slide (Figure S9, 7) and the water was removed to settle the scaffold onto the slide (Figure S9, 8). Next, the slide was dried using a Kimtech tissue (Figure S9, 9) and the scaffold was mounted using Mowiol (Figure S9, 10) as well as a glass coverslip (Figure S9, 11). The mounted samples were dried overnight at RT and then stored at 4 °C in the dark until imaging.

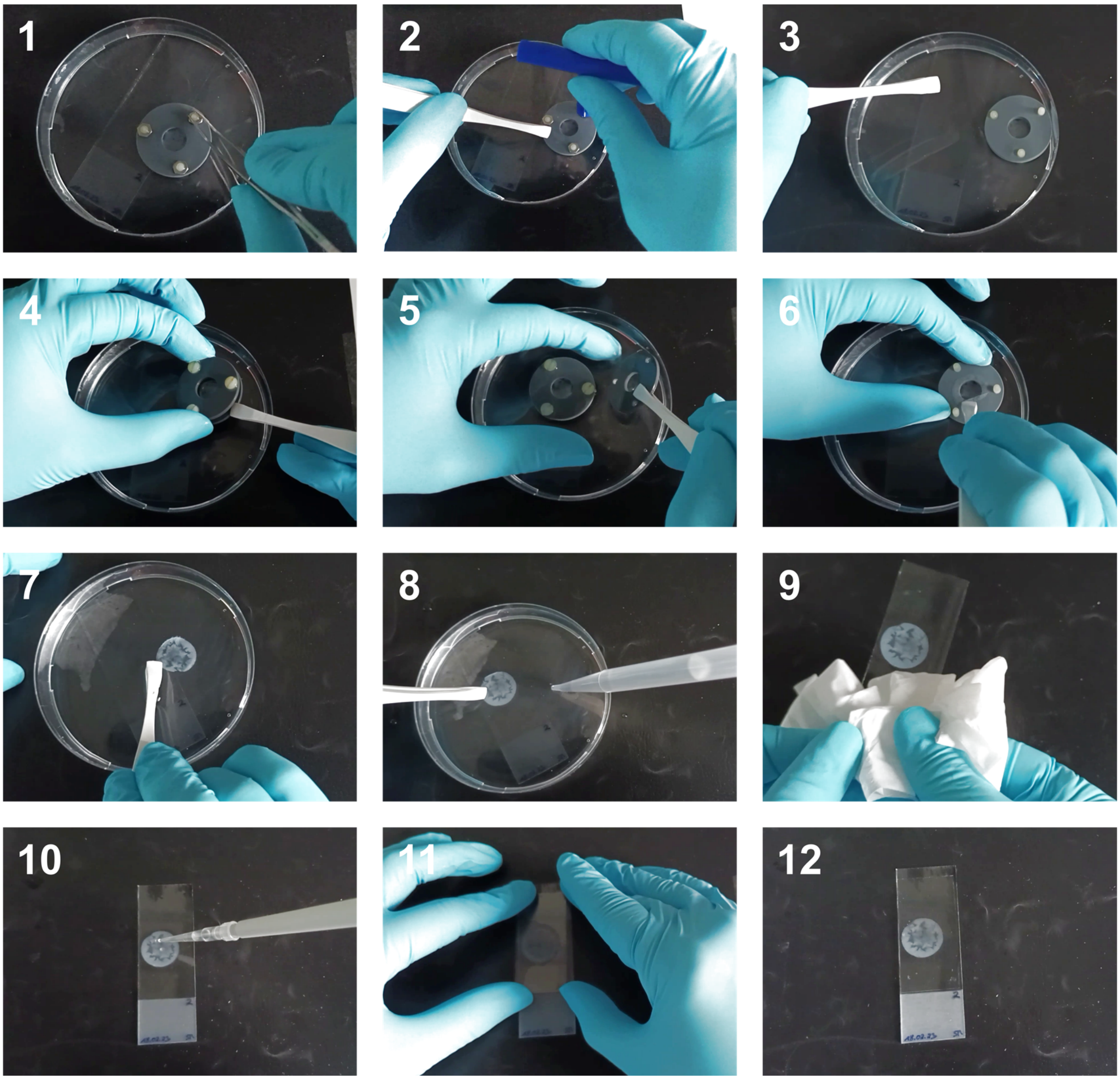

Figure S9. Mounting procedure for fibrinogen scaffolds after IF staining. (1) The clamped scaffolds are transferred into a petri dish containing a glass slide and ddH_2O. (2, 3) Disassembling of the nuts using a 3D printed hexagon spanner. (4, 5) Forceps are used to separate the PVC rings carefully. (6) The scaffold is released from the ring using forceps. (7) The floating scaffold is placed above the glass slide. (8) Removing of the water to settle the scaffold onto the slide. (9) Drying of the slide with a Kimtech tissue. (10, 11) Mounting of the scaffold with Mowiol and a glass coverslip. IF: immunofluorescence; PVC: polyvinyl chloride. A video of the detachment and clamping process is shown in supplementary video 8.

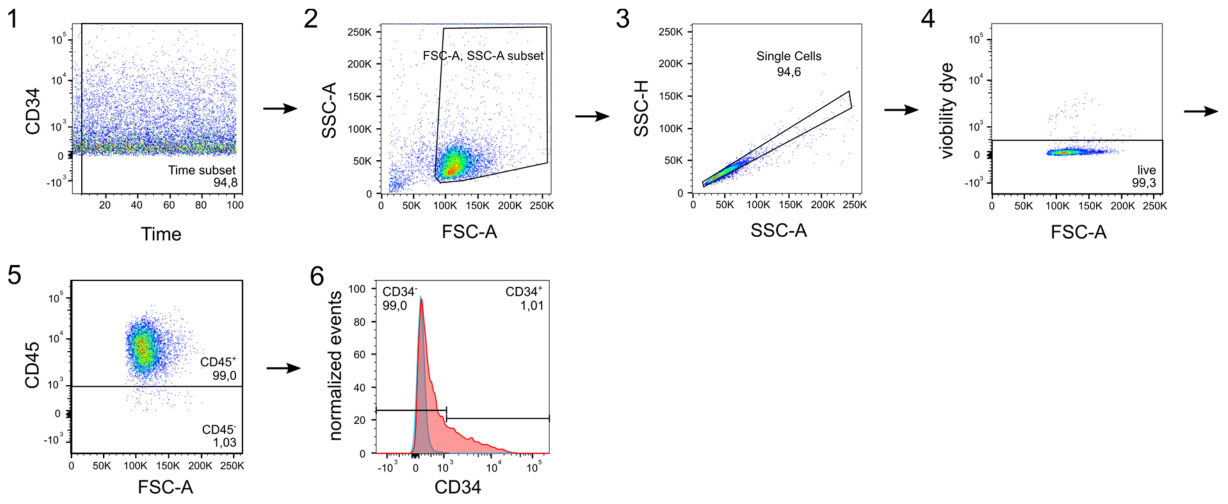

Figure S10. Gating strategy for the analysis of flow cytometry data of HSPCs after culture on free-standing fibrinogen scaffolds. (1) The initial seconds were eliminated as irregularities in the sample supply can occur in the beginning. (2) The cell trash was excluded from the analysis. (3) The signals of single cells align in a diagonal in the SSC-H vs. SSC-A plot, which enabled the elimination of doublets. (4) Based on a live/dead staining of a control sample, the dead cells in the experimental conditions could be detected and eliminated from the analysis. (5) A CD45 gate was implemented to identify $CD45^{+}$ hematopoietic cells, which selected to continue the analysis. (6) A 1% gate was set in the isotype control sample and the resulting gate was transferred to the sample staining to ensure that the analyzed signals were not generated by unspecific binding of the cells to the ABs. As a rule, 1% of unspecific binding was tolerated in the analysis. The gating strategy is exemplarily shown for the data of n1.

Table S2: List of antibodies and dyes purchased by Miltenyi Biotech used for flow cytometry.

Antibody or dye	Clone	Dilution	Cat.-No.; RRID	Lot.-No.
CD34 Antibody, Anti Human, APC, REAfinity™	REA1164	1:100	Cat#130 120 514; RRID: AB_2811337	5230205685
CD45 Antibody, Anti Human, PE, REAfinity™	REA747	1:50	Cat#130-110-770; RRID: AB_2658238	5220504068
REA Control Antibody (S), Human IgG1, APC, REAfinity™	REA293	1:50	Cat#130-113-434; RRID: AB_2733447	5231207777
REA Control Antibody (S), Human IgG1, PE, REAfinity™	REA293	1:50	Cat#130-113-438; RRID: AB_2733893	5231208953
REA Control Antibody (S), Human IgG1, VioGreen™, REAfinity™	REA293	1:50	Cat#130-113-444; RRID: AB_2734114	5220405581
Viobility Dye 405/520	–	1:100	Cat#130-109-814	5241010278

Table S3: List of antibodies and dyes used for IF staining.

Antibody or dye	Dilution and Incubation	Supplier	Cat.-No.; RRID	Lot.-No.
CD45 Primary Antibody, IgG2a, Rat, Monoclonal (Clone: YAML501.4)	1:500, overnight, 4 °C	Thermo Fisher Scientific	Cat# MA5 17687; RRID: AB_2539077	XG3656247
Vinculin Primary Antibody, IgG1, Mouse, Monoclonal (Clone: VLN01)	1:50, 1 h, RT	Thermo Fisher Scientific	Cat# MA 511690; RRID: AB_10976821	UL2902015
Goat Anti Rat IgG (H+L) Cross Adsorbed Secondary Antibody, Alexa Fluor™ 647	1:1000, 1 h, RT	Thermo Fisher Scientific	Cat# A 21247; RRID: AB_141778	2420724
Goat Anti Mouse IgG (H+L) Highly Cross Adsorbed Secondary Antibody, Alexa Fluor™ 647	1:1000, 1 h, RT	Thermo Fisher Scientific	Cat# A 21236; RRID: AB_2534071	2229182
Goat Anti Mouse IgG (H+L) Cross Adsorbed Secondary Antibody, Alexa Fluor™ 546	1:1000, 1 h, RT	Thermo Fisher Scientific	Cat# A 11003; RRID: AB_2534071	-
Phalloidin iFluor 555 Reagent	1:1000, 2 h, RT	abcam	Cat# ab176756	1015135-1
Phalloidin, Alexa Fluor™ 647	1:40, 2 h, RT	Thermo Fisher Scientific	Cat# A22287	2431325
DAPI (4',6 diamidino 2-phenylindole, dihydrochloride)	1:1000, 2 h, RT	Thermo Fisher Scientific	Cat# 62247	ZC4219251
CellTracker™ Green CMFDA Dye (CTG)	10 µM staining solution, 30 min, 37 °C and 5% CO_2	Thermo Fisher Scientific	Cat# C7025	2403702

For most of the samples, phalloidin conjugated to iFluor 555 was used to stain F-actin. However, F-actin staining in HMEC-1 cells on immobilized fibrinogen scaffolds using phalloidin conjugated to iFluor 555 was too weak to be visible as it was superimposed by the scaffold autofluorescence. For this reason, phalloidin conjugated to Alexa Fluor™ 647 was used to stain F-actin in these samples.

Table S4: Composition of cell culture media for culture on free-standing and immobilized fibrinogen scaffolds.

Type of medium	Composition of medium	
**Double co-culture medium (DCM)**	10% (v/v)	FBS (Merck, Darmstadt, Germany)
	5% (v/v)	ROTI®Cell Glutamine solution (Carl Roth, Karlsruhe, Germany)
	1% (v/v)	P/S (Merck, Darmstadt, Germany)
		MCDB 131 Medium (PAN Biotech, Aidenbach, Germany)
**Triple co-culture medium (TCM)**	10% (v/v)	FBS (Merck, Darmstadt, Germany)
	2% (v/v)	BSA (50 mg/mL stock solution in ddH_2O, Merck, Darmstadt, Germany)
	1% (v/v)	P/S/G (Penicillin-streptomycin-glutamine (100X), Thermo Fisher Scientific, Waltham, MA, USA)
	1% (v/v)	ITS-X (Insulin-Transferrin-Selenium-Ethanolamine (100X), Thermo Fisher Scientific, Waltham, MA, USA)
	1% (v/v)	HEPES (4-(2-hydroxyethyl)-1-piperazineethanesulfonic acid, 1 mM stock solution, Merck, Darmstadt, Germany)
	1% (v/v)	Cytokine Mix E (for HPC Expansion Medium XF, PromoCell, Heidelberg, Germany)
	0.04% (v/v)	EGF (0.4 µL/mL; from Supplement Mix for Endothelial Cell Growth Medium 2, PromoCell, Heidelberg, Germany)
		Iscove's Modified Dulbecco's Medium (Thermo Fisher Scientific, Waltham, MA, USA)
